# Supplementary material for: Conformity to social norm interventions is not amplified in tighter nations
Source: Commun Psychol. 2026 Mar 5;4:68. doi: 10.1038/s44271-026-00429-4 (PMC13083840; doi:10.1038/s44271-026-00429-4)
Supplement: Supplementary file 2 — Supplemental Materials [file 44271_2026_429_MOESM2_ESM.pdf]

Conformity to social norm interventions is not amplified in tighter nations

Supplementary Online Materials

Descriptive Statistics

Table S1 – Correlations

Table S2 – National Sample Sizes

Table S3 – Descriptive Statistics by Country

Power Analyses

Cultural Tightness Measures Information

Figure S1 – Correlations Between Cultural Tightness and Individualism-Collectivism

Figure S2 – Correlations Between the two Cultural Tightness Measures

Main Hypothesized Models with and without Covariates

Table S4 – Models for Belief in Climate Change

Table S5 – Models for Climate Mitigation Policy Support

Table S6 – Models for Social Media Post Sharing Intention

Table S7 – Models for Tree Planting Task

Table S8 – Models for All Outcomes Without Political Orientation

Main Hypothesized Models Clustering by Country Standard Errors

Table S9 – Models for Belief in Climate Change

Table S10 – Models for Climate Mitigation Policy Support

Table S11 – Models for Social Media Post Sharing Intention

Table S12 – Models for Tree Planting Task

Comparisons Between Experimental Conditions

Pluralistic Ignorance Supplemental Analyses

Table S13 – Pluralistic Ignorance Exploratory Analyses – PI ⊗Estimates as covariate

Table S14 – Pluralistic Ignorance Under- vs. Overestimation of the Norm

Table S15 – Pluralistic Ignorance Norm Percentage

Table S16 – Pluralistic Ignorance Exploratory Analyses – PI Area (Region vs. Country)

Models with an Alternative Cultural Tightness Measure (Uz, 2015)

Table S17 – Models for Belief in Climate Change

Table S18 – Models for Climate Mitigation Policy Support

Table S19 – Models for Social Media Post Sharing Intention

Table S20 – Models for Tree Planting Task

Bayesian Analysis Testing for Support of the Null Hypothesis

[Experimental Effects in the US]

Table S21 – Experimental Intervention Effects in the US

[Additional National-Level Covariates]

Table S22 – Models for Belief in Climate Change

Table S23 – Models for Climate Mitigation Policy Support

Table S24 – Models for Social Media Post Sharing Intention

Table S25 – Models for Tree Planting Task

[Representativeness Checks]

Table S26 – Models for Belief in Climate Change

Table S27 – Models for Climate Mitigation Policy Support

Table S28 – Models for Social Media Post Sharing Intention

Table S29 – Models for Tree Planting Task

[Cumulative CO_2 Emissions Median Split]

Table S30 – Models for Belief in Climate Change

Table S31 – Models for Climate Mitigation Policy Support

Table S32 – Models for Social Media Post Sharing Intention

Table S33 – Models for Tree Planting Task

[GDP per Capita Median Split]

Table S34 – Models for Belief in Climate Change

Table S35 – Models for Climate Mitigation Policy Support

Table S36 – Models for Social Media Post Sharing Intention

Table S37 – Models for Tree Planting Task

[Cumulative CO_2 Emissions Interaction]

Table S38 – Models Testing the 3-way Interaction

[Correlations Between National-Level Variables]

Table S39 – Correlations Between National-Level Variables

Descriptive Statistics

The following tables contain correlations between the main individual-level covariates and the dependent variables for the entire sample (Table S1), sample size and cultural tightness of each country (Table S2), and the descriptive statistics of these variables divided by country (Table S3).

We find that, although individual-level covariates correlate with each other, there are no strong correlations with the dependent variables (see Table S1). In fact, even across outcomes, the only strong correlation is between belief in climate change and support for climate change policies — our two attitude outcome measures.

Table S1.
Correlations Between Individual-Level Covariates and Dependent Variables.

	Age	Political orientation	Education level	Income	Belief in CC	Policy Support	Social Media Post	Tree Planting
**Political orientation**	.07***	-						
**Education level**	.00	.01	-					
**Income**	.00	.03***	.23***	-				
**Belief in CC**	-.15***	-.21***	.04***	.00	-			
**Policy Support**	-.09***	-.15***	.07***	.02	.69***	-		
**Social Media Post**	-.06***	-.11***	.11***	.04***	.23***	.26***	-	
**Tree Planting**	.14***	-.07***	.03***	.01	.13***	.11***	.05***	-
**Cultural tightness**	-.12***	.13***	.05***	-.07***	.06***	.05***	.18***	-.03***

Table S2.

National Sample Sizes and Estimates of Cultural Tightness.

**Country**	**Sample size of the current study**	**Sample size from full ManyLabs data (Vlasceanu et al., 2024)**	**Cultural tightness scores from Eriksson et al. (2021)**
Algeria	177	528	2.46
Armenia	170	492	2.07
Australia *	333	979	1.90
Austria *	177	502	2.10
Brazil *	447	1261	1.69
Canada †	396	1161	1.83
Chile *	669	1992	1.68
China	307	896	2.05
Czech Republic	187	547	1.63
Ecuador *	221	679	1.82
Finland *	267	625	1.75
Germany *	538	1545	2.03
Ghana *	174	522	2.93
Greece *	256	597	1.71
India	241	688	2.48
Ireland	262	753	1.80
Israel *	459	1384	1.66
Italy *	540	1584	1.87
Japan †	507	1455	2.09
Kenya *	96	409	2.36
Latvia *	187	485	1.88
Mexico *	172	490	1.69
Netherlands †	652	1864	1.59
Nigeria *	529	1513	2.26
Peru *	150	405	1.70
Poland †	819	2346	1.70
Portugal	167	499	2.00
Russia †	504	1435	1.67

Saudi Arabia	153	489	2.40
Singapore	171	500	2.24
Slovakia *	354	1027	1.71
South Korea *	219	639	2.09
Spain †	179	544	1.71
Sri Lanka	146	413	2.32
Sweden *	815	2393	2.20
Thailand	185	586	2.13
Turkey †	246	706	2.17
UK †	661	1975	1.77
Ukraine	183	496	1.74
United Arab Emirates *	189	554	2.33
USA *	2837	8253	1.82
Vietnam	128	383	2.19

Note: * indicates all samples representative on at least one demographic dimension; † indicates at least one sample representative and at least one sample non-representative. For further information about the samples and representativeness, refer to Table 1 of Vlasceanu and colleagues (2024).

Table S3.
Descriptive Statistics for Individual-Level Covariates and Dependent Variables by Country.

	Age	Gender			Political orientation	Education level	Income	Belief in Climate Change	Policy Support	Social Media Post	Tree Planting
		Male	Female	Non-binary or Other							
**Algeria (*N*=177)**	33.0 (9.50)	125 (70.6%)	51 (28.8%)	0 (0%)	60.2 (18.9)	2.94 (0.665)	3.67 (2.24)	80.2 (21.8)	75.3 (15.9)	0.700 (0.460)	3.95 (3.30)
**Armenia (*N*=170)**	30.6 (13.1)	66 (38.8%)	104 (61.2%)	0 (0%)	61.8 (22.3)	2.87 (0.696)	3.91 (1.59)	83.5 (17.6)	68.2 (17.1)	0.359 (0.481)	3.13 (3.23)
**Australia (*N*=333)**	46.6 (15.9)	137 (41.1%)	194 (58.3%)	2 (0.6%)	49.6 (19.7)	2.86 (0.634)	5.21 (1.45)	75.8 (25.3)	69.2 (19.4)	0.408 (0.493)	4.77 (3.25)
**Austria (*N*=177)**	38.3 (13.2)	83 (46.9%)	93 (52.5%)	1 (0.6%)	50.8 (20.4)	2.56 (0.923)	4.03 (1.37)	74.2 (25.8)	67.4 (21.0)	0.259 (0.440)	3.18 (3.30)
**Brazil (*N*=447)**	38.0 (13.6)	210 (47.0%)	235 (52.6%)	1 (0.2%)	59.1 (28.4)	2.80 (0.775)	3.96 (1.58)	86.7 (18.6)	77.1 (17.5)	0.683 (0.466)	4.36 (3.40)
**Canada (*N*=396)**	27.7 (14.7)	118 (29.8%)	265 (66.9%)	10 (2.5%)	40.3 (22.5)	2.76 (0.515)	5.36 (1.61)	83.1 (21.5)	70.5 (17.1)	0.304 (0.461)	4.21 (3.25)
**Chile (*N*=669)**	43.4 (19.0)	77 (11.5%)	62 (9.3%)	1 (0.1%)	45.8 (22.8)	2.88 (0.599)	6.41 (2.08)	87.7 (18.3)	78.3 (15.6)	0.707 (0.455)	4.53 (3.07)
**China (*N*=307)**	28.0 (7.26)	116 (37.8%)	190 (61.9%)	0 (0%)	54.5 (19.9)	3.12 (0.377)	6.97 (1.63)	84.0 (12.0)	75.5 (12.7)	0.764 (0.425)	5.07 (3.08)
**Czech Republic (*N*=187)**	26.8 (12.3)	50 (26.7%)	134 (71.7%)	1 (0.5%)	45.6 (19.4)	2.98 (0.691)	5.65 (2.04)	81.5 (24.1)	65.6 (18.9)	0.178 (0.384)	4.44 (3.35)
**Ecuador (*N*=221)**	32.8 (10.3)	114 (51.6%)	101 (45.7%)	3 (1.4%)	56.2 (19.5)	2.88 (0.598)	3.13 (2.12)	85.9 (20.2)	72.8 (17.1)	0.871 (0.336)	3.51 (3.21)

**Finland (*N*=267)**	41.1 (14.9)	101 (37.8%)	165 (61.8%)	0 (0%)	51.2 (23.7)	2.79 (0.693)	4.20 (1.35)	73.2 (24.7)	62.6 (19.6)	0.335 (0.473)	3.13 (3.28)
**Germany (*N*=538)**	47.3 (15.4)	275 (51.1%)	258 (48.0%)	1 (0.2%)	48.5 (17.1)	2.48 (0.920)	3.93 (1.39)	77.6 (25.1)	65.6 (20.0)	0.255 (0.436)	4.08 (3.42)
**Ghana (*N*=174)**	29.5 (8.43)	103 (59.2%)	68 (39.1%)	0 (0%)	61.4 (22.4)	2.91 (0.557)	1.86 (1.69)	86.5 (16.6)	77.0 (15.8)	0.818 (0.388)	3.97 (3.48)
**Greece (*N*=256)**	40.1 (12.2)	146 (57.0%)	108 (42.2%)	0 (0%)	51.7 (20.6)	2.96 (0.655)	2.71 (1.19)	73.6 (25.3)	67.1 (18.2)	0.641 (0.481)	4.08 (3.31)
**India (*N*=241)**	26.8 (8.47)	157 (65.1%)	78 (32.4%)	2 (0.8%)	61.9 (25.4)	3.22 (0.553)	4.46 (2.26)	86.3 (13.3)	77.9 (11.7)	0.786 (0.411)	2.15 (2.99)
**Ireland (*N*=262)**	26.8 (9.08)	130 (49.6%)	124 (47.3%)	3 (1.1%)	43.6 (23.8)	3.09 (0.688)	4.83 (1.49)	81.6 (21.6)	72.0 (17.2)	0.610 (0.489)	2.61 (3.06)
**Israel (*N*=459)**	38.6 (12.7)	240 (52.3%)	217 (47.3%)	1 (0.2%)	54.4 (24.7)	2.95 (0.580)	4.38 (1.79)	66.9 (28.5)	66.0 (19.2)	0.287 (0.453)	4.16 (3.34)
**Italy (*N*=540)**	35.5 (14.0)	253 (46.9%)	249 (46.1%)	6 (1.1%)	37.1 (24.2)	2.89 (0.783)	3.48 (1.22)	86.9 (19.0)	72.0 (16.4)	0.566 (0.496)	5.06 (3.12)
**Japan (*N*=507)**	45.8 (11.2)	319 (62.9%)	181 (35.7%)	0 (0%)	51.9 (15.1)	2.85 (0.523)	4.11 (1.81)	73.9 (21.9)	58.5 (16.0)	0.193 (0.395)	4.72 (3.32)
**Kenya (*N*=96)**	32.3 (14.4)	52 (54.2%)	44 (45.8%)	0 (0%)	NA (NA)	2.81 (0.586)	2.48 (1.85)	89.1 (13.0)	72.1 (16.6)	0.936 (0.247)	2.09 (2.84)
**Latvia (*N*=187)**	52.2 (14.6)	51 (27.3%)	131 (70.1%)	3 (1.6%)	53.3 (20.1)	2.99 (0.740)	2.36 (1.43)	66.6 (29.9)	61.7 (19.4)	0.104 (0.306)	4.07 (3.16)
**Mexico (*N*=172)**	37.6 (13.4)	88 (51.2%)	83 (48.3%)	1 (0.6%)	58.5 (20.5)	2.81 (0.667)	6.48 (2.14)	88.6 (13.7)	75.3 (16.3)	0.877 (0.330)	4.01 (3.31)
**Netherlands (*N*=652)**	39.1 (17.9)	253 (38.8%)	393 (60.3%)	4 (0.6%)	47.1 (21.6)	2.70 (0.555)	4.15 (1.40)	76.1 (24.1)	69.9 (18.6)	0.185 (0.389)	3.72 (3.27)

**Nigeria (*N*=529)**	32.3 (9.70)	318 (60.1%)	204 (38.6%)	0 (0%)	65.9 (21.7)	3.13 (0.513)	3.72 (2.14)	86.7 (15.8)	76.5 (15.8)	0.852 (0.356)	4.55 (3.39)
**Peru (*N*=150)**	24.3 (8.10)	63 (42.0%)	83 (55.3%)	2 (1.3%)	55.5 (17.5)	2.87 (0.515)	3.35 (2.20)	87.4 (15.4)	71.6 (17.0)	0.807 (0.397)	3.67 (3.28)
**Poland (*N*=819)**	43.0 (17.3)	343 (41.9%)	468 (57.1%)	5 (0.6%)	46.0 (23.5)	2.57 (0.598)	2.53 (1.33)	78.1 (23.8)	68.5 (18.4)	0.328 (0.470)	4.74 (3.27)
**Portugal (*N*=167)**	27.4 (8.07)	91 (54.5%)	75 (44.9%)	1 (0.6%)	42.4 (20.1)	2.87 (0.428)	3.08 (1.18)	91.9 (12.7)	74.4 (13.9)	0.370 (0.485)	4.38 (3.12)
**Russia (*N*=504)**	29.6 (11.7)	195 (38.7%)	298 (59.1%)	1 (0.2%)	48.1 (18.9)	2.87 (0.564)	4.48 (1.60)	73.2 (24.1)	63.4 (17.8)	0.221 (0.416)	3.77 (3.32)
**Saudi Arabia (*N*=153)**	33.8 (8.72)	84 (54.9%)	69 (45.1%)	0 (0%)	62.0 (19.3)	3.08 (0.602)	3.36 (1.72)	71.7 (24.4)	66.7 (19.7)	0.772 (0.421)	4.25 (3.38)
**Singapore (*N*=171)**	36.5 (11.9)	90 (52.6%)	80 (46.8%)	0 (0%)	56.0 (18.4)	3.07 (0.561)	5.34 (1.66)	80.7 (16.6)	70.7 (16.2)	0.721 (0.450)	3.23 (3.20)
**Slovakia (*N*=354)**	43.8 (15.2)	145 (41.0%)	206 (58.2%)	2 (0.6%)	51.5 (19.0)	2.74 (0.670)	4.50 (1.65)	75.6 (24.4)	63.0 (17.9)	0.298 (0.458)	5.45 (3.14)
**South Korea (*N*=219)**	43.6 (14.0)	96 (43.8%)	121 (55.3%)	0 (0%)	54.1 (16.8)	2.95 (0.571)	4.39 (1.21)	80.8 (18.2)	70.7 (16.3)	0.743 (0.439)	5.09 (3.20)
**Spain (*N*=179)**	45.1 (15.3)	88 (49.2%)	89 (49.7%)	0 (0%)	40.2 (25.5)	3.21 (0.797)	3.26 (1.28)	77.9 (26.1)	71.9 (20.9)	0.474 (0.502)	4.74 (3.28)
**Sri Lanka (*N*=146)**	27.0 (7.29)	106 (72.6%)	35 (24.0%)	1 (0.7%)	59.8 (19.9)	3.00 (0.544)	2.58 (1.92)	84.0 (16.8)	78.0 (12.1)	0.632 (0.485)	4.03 (3.36)
**Sweden (*N*=815)**	41.2 (15.5)	362 (44.4%)	443 (54.4%)	5 (0.6%)	52.7 (22.9)	2.59 (0.638)	4.96 (1.97)	72.2 (26.0)	63.6 (20.7)	0.509 (0.500)	3.37 (3.37)
**Thailand (*N*=185)**	36.9 (11.4)	103 (55.7%)	77 (41.6%)	5 (2.7%)	61.6 (23.9)	2.89 (0.708)	2.68 (1.76)	76.8 (24.8)	71.7 (21.9)	0.779 (0.416)	3.84 (3.36)

**Turkey (*N*=246)**	32.3 (12.6)	82 (33.3%)	160 (65.0%)	2 (0.8%)	44.8 (28.2)	2.88 (0.568)	3.62 (1.76)	89.4 (15.8)	80.3 (16.7)	0.421 (0.495)	4.62 (3.31)
**Ukraine (*N*=183)**	31.1 (11.2)	54 (29.5%)	123 (67.2%)	2 (1.1%)	47.5 (20.3)	3.42 (0.660)	4.04 (2.48)	79.8 (22.1)	76.2 (14.5)	0.347 (0.478)	4.23 (3.27)
**United Arab Emirates (*N*=189)**	35.2 (8.65)	99 (52.4%)	90 (47.6%)	0 (0%)	60.7 (20.1)	3.15 (0.485)	4.24 (2.04)	77.6 (22.5)	69.9 (18.1)	0.806 (0.397)	4.84 (3.29)
**United Kingdom (*N*=680)**	39.1 (16.1)	266 (39.1%)	396 (58.2%)	10 (1.5%)	48.1 (23.5)	2.95 (0.671)	4.23 (1.89)	78.0 (22.4)	70.2 (18.8)	0.526 (0.500)	3.70 (3.27)
**United States (*N*=2837)**	46.0 (16.4)	1331 (46.9%)	1453 (51.2%)	28 (1.0%)	55.6 (26.7)	2.89 (0.652)	4.51 (1.64)	68.5 (30.3)	64.0 (24.0)	0.582 (0.493)	3.98 (3.37)
**Vietnam (*N*=128)**	22.1 (5.82)	34 (26.6%)	90 (70.3%)	2 (1.6%)	59.8 (21.4)	2.90 (0.469)	2.42 (1.84)	88.6 (13.2)	77.7 (14.1)	0.763 (0.427)	4.20 (3.30)
**Overall (*N*=16089)**	38.8 (15.7)	7214 (44.8%)	8088 (50.3%)	106 (0.7%)	52.2 (23.7)	2.86 (0.664)	4.18 (1.94)	77.6 (24.6)	69.0 (19.7)	0.522 (0.500)	4.11 (3.34)

Note: [1] There was no political ideology data collected from Kenya. All reported measures are means and standard errors, except for gender, which reported counts and percentages.

Power Analysis

A Priori Sensitivity

To estimate the minimum detectable effect size (MDES) with this sample, we conducted an *a priori* sensitivity analysis with a random 10% of the data from the ManyLabs project. Using the SIMR package for R (Green & MacLeod, 2016), we ran a Monte Carlo simulation testing H1a (without covariates) to get estimates of covariance between factors in the model and the structure of the nested data, while expanding the observations in this smaller sample to increase the number of countries and estimated sample size within each country to match our final sample size. By iteratively increasing/decreasing the observed effect of the interaction we were able to determine the MDES for 80% power. After running 10,000 simulations, we identified an MDES of η_p^2 = .0005 for 80% power and η_p^2 = .0008 for 95% power.

Cultural Tightness Measures Information

Cultural Tightness Measures Specifications

Other published studies have measured cultural tightness using the same measure as Eriksson and colleagues (2021), which would potentially allow us to include more countries from the ManyLabs dataset in our final analysis. However, the measure of cultural tightness is rescaled in each study in comparison to the other measures assessed in that study. So, while the same questions were asked in other studies, the reported levels of cultural tightness are not comparable across studies. As a result, we are unable to combine Eriksson and colleagues' (2021) data with other existing datasets.

Cultural Tightness Correlations

The following figures represent the correlations of the cultural tightness measure we used in the main text (Erikson et al., 2021) with the Individualism-Collectivism measure we used as a control (Figure S1), and with the alternative cultural tightness measure (Uz, 2015) we used in the supplemental analyses (see Tables S11-S14).

Figure S1.

Correlations Between Cultural Tightness and Individualism-Collectivism.

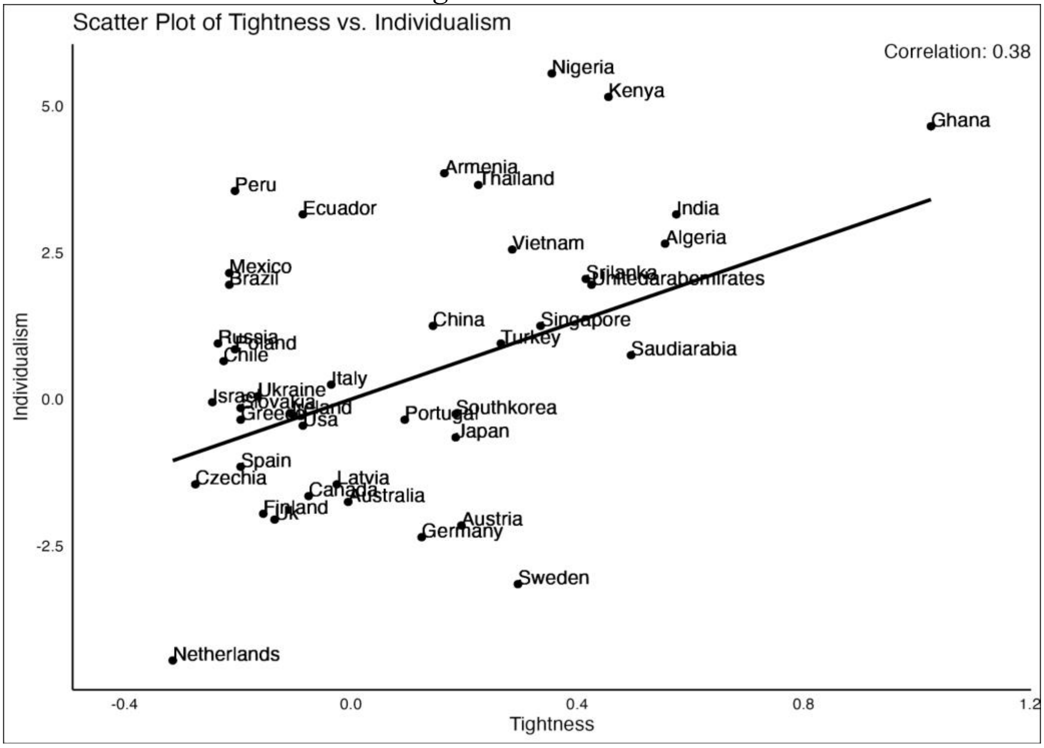

Note: The cultural tightness measure used here is the same as the one reported in the main text (Erikson et al., 2021)

Figure S2.

Correlations Between Cultural Tightness Measures.

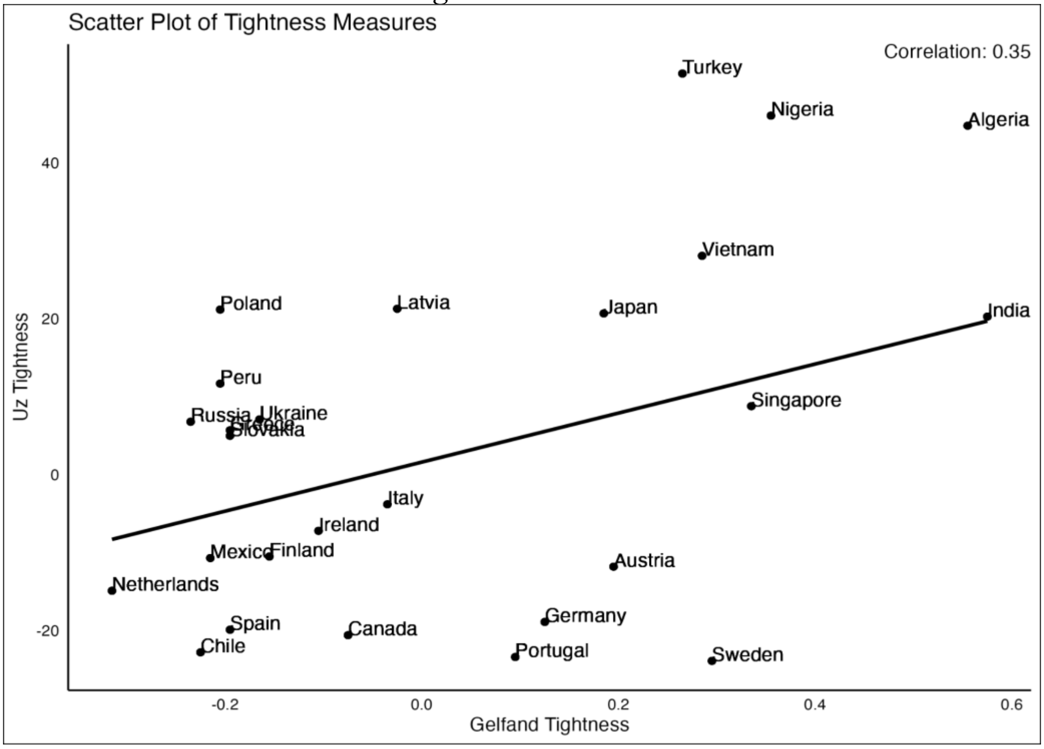

Note. Correlation plot between Gelfand's and Uz's Cultural Tightness measures using countries represented in both datasets (N=26).

Main Hypothesized Models With and Without Covariates

The following tables represent the results of the models reported in main text but with different sets of controls. Each outcome is represented in a different table: Belief in Climate Change in Table S4, Climate Mitigation Policy Support in Table S5, Social Media Post in Table S6, Tree Planting Task in Table S7. For each of the tables, Model 1 represents the model without any covariates. Model 2 represents the model with all covariates, including national estimates of cultural individualism-collectivism.

Belief in Climate Change

Both with and without covariates, the interaction of cultural tightness with the comparison between Pluralistic Ignorance and Control conditions is significantly negative. As in the main text, even when controlling for individualism-collectivism, the interaction of cultural tightness with the comparison between Work-Together Norm and Control conditions was significant, though it did not reach statistical significance without any controls present.

Table S4.

Predicting Belief in Climate Change Without Covariates (Model 1) and With Individualism (Model 2).

	**Model 1**	**Model 2**
Predictors	*Estimates*	*Estimates*
Intercept	79.59 ***	76.50 ***
	(77.54 – 81.64)	(74.75 – 78.24)
Cultural Tightness	5.31	2.78
	(-1.71 – 12.32)	(-3.46 – 9.03)
Dynamic Norm	1.00	1.10
	(-0.17 – 2.16)	(-0.03 – 2.23)
Work-together Norm	-0.24	-0.15
	(-1.41 – 0.92)	(-1.29 – 0.98)
Pluralistic Ignorance	0.82	0.96
	(-0.34 – 1.98)	(-0.16 – 2.09)
Dynamic Norm * Tightness	-0.71	-0.21
	(-5.38 – 3.97)	(-4.74 – 4.32)
Work-together Norm * Tightness	4.64	4.91 *
	(-0.07 – 9.35)	(0.35 – 9.48)
Pluralistic Ignorance * Tightness	-5.47 *	-5.39 *
	(-10.17 – -0.76)	(-9.95 – -0.83)
Age		-0.09 ***
		(-0.12 – -0.06)
Gender: Female		4.04 ***
		(3.23 – 4.86)
Gender: Non-binary/other		4.43
		(-0.74 – 9.60)
Education		0.79 *
		(0.15 – 1.44)
Income		0.44 ***
		(0.19 – 0.68)
Political orientation		-0.22 ***
		(-0.24 – -0.20)
Emissions per capita		0.37
		(-0.28 – 1.03)
GDP per capita		-0.18
		(-0.97 – 0.61)
Individualism		0.19 ***
		(0.11 – 0.27)
**Random Effects**		
σ^2	553.32	519.61
τ_{00}	34.63 Country	19.35 Country
ICC	0.06	0.04
N	41 Country	41 Country
Observations	12760	12760
Marginal R^2 / Conditional R^2	0.004 / 0.063	0.082 / 0.115

* *p<0.05* ** *p<0.01* *** *p<0.001*

Climate Mitigation Policy Support

Both with and without covariates, the interaction of cultural tightness with the comparison between Pluralistic Ignorance and Control conditions is significantly negative. Neither of the other two interactions reached statistical significance with or without any controls present.

Table S5.
Predicting Climate Mitigation Policy Support without Covariates (Model 1) and with Individualism (Model 2).

	**Model 1**	**Model 2**
Predictors	*Estimates*	*Estimates*
Intercept	70.24 *** (68.50 – 71.98)	68.84 *** (67.28 – 70.39)
Cultural Tightness	6.62 * (0.68 – 12.57)	4.12 (-1.41 – 9.66)
Dynamic Norm	1.19 * (0.25 – 2.13)	1.29 ** (0.37 – 2.22)
Work-together Norm	0.16 (-0.78 – 1.11)	0.20 (-0.73 – 1.13)
Pluralistic Ignorance	0.59 (-0.34 – 1.53)	0.70 (-0.23 – 1.62)
Dynamic Norm * Tightness	-2.06 (-5.84 – 1.72)	-1.74 (-5.46 – 1.98)
Work-together Norm * Tightness	2.12 (-1.69 – 5.93)	2.36 (-1.39 – 6.11)
Pluralistic Ignorance * Tightness	-4.78 * (-8.58 – -0.97)	-4.68 * (-8.42 – -0.94)
Age		0.00 (-0.02 – 0.02)
Gender: Female		1.48 *** (0.81 – 2.15)
Gender: Non-binary/other		3.71 (-0.56 – 7.97)
Education		1.38 *** (0.85 – 1.91)
Income		0.40 *** (0.20 – 0.60)
Political orientation		-0.14 *** (-0.15 – -0.12)
Emissions per capita		0.46 (-0.13 – 1.05)
GDP per capita		-0.60 (-1.32 – 0.11)
Individualism		0.16 *** (0.08 – 0.23)
**Random Effects**		
σ^2	361.31	349.31
τ_{00}	25.55 Country	16.15 Country
ICC	0.07	0.04
N	41 Country	41 Country
Observations	12722	12722
Marginal R^2 / Conditional R^2	0.006 / 0.072	0.056 / 0.098

* $p<0.05$ ** $p<0.01$ *** $p<0.001$

Social Media Post Sharing Intention

Both with and without covariates, none of the three interactions reached statistical significance.

Table S6.

Predicting Social Media Sharing Intentions Without Covariates (Model 1) and With Collectivism (Model 2).

	**Model 1**	**Model 2**
Predictors	*Estimates*	*Estimates*
Intercept	0.50 ***	0.51 ***
	(0.43 – 0.57)	(0.45 – 0.56)
Cultural Tightness	0.35 **	0.12
	(0.12 – 0.58)	(-0.07 – 0.32)
Dynamic Norm	0.07 ***	0.07 ***
	(0.04 – 0.09)	(0.04 – 0.09)
Work-together Norm	0.05 ***	0.05 ***
	(0.03 – 0.08)	(0.03 – 0.08)
Pluralistic Ignorance	0.02	0.02
	(-0.01 – 0.04)	(-0.01 – 0.04)
Dynamic Norm * Tightness	0.01	0.01
	(-0.08 – 0.11)	(-0.09 – 0.11)
Work-together Norm * Tightness	0.01	0.01
	(-0.09 – 0.11)	(-0.09 – 0.11)
Pluralistic Ignorance * Tightness	-0.03	-0.03
	(-0.13 – 0.07)	(-0.13 – 0.07)
Age		-0.00 ***
		(-0.00 – -0.00)
Gender: Female		-0.05 ***
		(-0.07 – -0.03)
Gender: Non-binary/other		-0.10
		(-0.22 – 0.01)
Education		0.04 ***
		(0.02 – 0.05)
Income		0.00
		(-0.00 – 0.01)
Political orientation		0.00 ***
		(0.00 – 0.00)
Emissions per capita		0.01
		(-0.01 – 0.03)
GDP per capita		-0.03 *
		(-0.06 – -0.00)
Individualism		0.01 ***
		(0.00 – 0.01)
**Random Effects**		
σ^2	0.21	0.20
τ_{00}	0.04 Country	0.02 Country
ICC	0.17	0.10
N	41 Country	41 Country
Observations	9794	9794
Marginal R^2 / Conditional R^2	0.034 / 0.202	0.108 / 0.199

* *p<0.05* ** *p<0.01* *** *p<0.001*

Tree Planting Task

The interaction of cultural tightness with the comparison between Pluralistic Ignorance and Control conditions is significantly negative in the model without covariates. However, none of the other interactions reached statistical significance.

Table S7.

Predicting the Tree Planting Task without Covariates (Model 1) and with Collectivism (Model 2).

	**Model 1**	**Model 2**
Predictors	*Estimates*	*Estimates*
Intercept	4.39 ***	4.21 ***
	(4.14 – 4.64)	(3.96 – 4.45)
Cultural Tightness	-0.13	-0.14
	(-1.00 – 0.75)	(-1.02 – 0.74)
Dynamic Norm	-0.08	-0.08
	(-0.24 – 0.08)	(-0.23 – 0.08)
Work-together Norm	-0.38 ***	-0.37 ***
	(-0.54 – -0.22)	(-0.53 – -0.21)
Pluralistic Ignorance	-0.11	-0.11
	(-0.27 – 0.06)	(-0.27 – 0.05)
Dynamic Norm * Tightness	-0.11	-0.03
	(-0.76 – 0.54)	(-0.67 – 0.61)
Work-together Norm * Tightness	-0.17	-0.10
	(-0.83 – 0.48)	(-0.75 – 0.55)
Pluralistic Ignorance * Tightness	-0.70 *	-0.59
	(-1.36 – -0.04)	(-1.23 – 0.06)
Age		0.04 ***
		(0.03 – 0.04)
Gender: Female		0.48 ***
		(0.36 – 0.59)
Gender: Non-binary/other		0.42
		(-0.31 – 1.15)
Education		0.06
		(-0.03 – 0.15)
Income		0.02
		(-0.01 – 0.06)
Political orientation		-0.01 ***
		(-0.01 – -0.01)
Emissions per capita		-0.10 *
		(-0.19 – -0.01)
GDP per capita		0.03
		(-0.08 – 0.14)
Individualism		0.01
		(-0.00 – 0.02)
**Random Effects**		
σ^2	10.77	10.40
τ_{00}	0.50 Country	0.38 Country
ICC	0.04	0.04
N	41 Country	41 Country
Observations	12769	12769
Marginal R^2 / Conditional R^2	0.003 / 0.047	0.047 / 0.081

* *p<0.05*   ** *p<0.01*   *** *p<0.001*

Excluding Political Orientation

The patterns of significance remain somewhat consistent with the main text here, with negative interactions for the comparison between Pluralistic Ignorance and Control conditions interacting with Tightness to predict Belief in Climate Change and Policy Support. However, the interaction of Tightness with the Work Together Norm condition (compared to control) was no longer significant in predicting Belief in Climate Change, and the interaction of Tightness with the Pluralistic Ignorance Norm condition (compared to control) was no longer significant in predicting Tree Planting.

Table S8.
Conditional and Interactive Effects on All Outcomes While Controlling for Covariates Without Political Orientation

	Belief in Climate Change	Policy Support	Social Media Post	Tree Planting
Predictors	*Estimates*	*Estimates*	*Estimates*	*Estimates*
Intercept	76.96 *** (74.91 – 79.01)	69.30 *** (67.55 – 71.04)	0.54 *** (0.47 – 0.60)	4.22 *** (3.97 – 4.47)
Cultural Tightness	5.11 (-1.91 – 12.13)	6.54 * (0.57 – 12.51)	0.28 * (0.06 – 0.50)	-0.07 (-0.92 – 0.78)
Dynamic Norm	1.11 (-0.04 – 2.27)	1.30 ** (0.36 – 2.23)	0.07 *** (0.04 – 0.09)	-0.07 (-0.23 – 0.08)
Work-together Norm	-0.16 (-1.32 – 0.99)	0.20 (-0.74 – 1.14)	0.05 *** (0.03 – 0.08)	-0.37 *** (-0.53 – -0.21)
Pluralistic Ignorance	0.93 (-0.23 – 2.08)	0.67 (-0.26 – 1.61)	0.02 (-0.01 – 0.04)	-0.11 (-0.27 – 0.05)
Age	-0.11 *** (-0.14 – -0.08)	-0.01 (-0.04 – 0.01)	-0.00 *** (-0.00 – -0.00)	0.04 *** (0.03 – 0.04)
Gender: Female	4.77 *** (3.94 – 5.60)	1.92 *** (1.25 – 2.60)	-0.05 *** (-0.07 – -0.03)	0.51 *** (0.40 – 0.63)
Gender: Non-binary/other	9.37 *** (4.10 – 14.65)	6.83 ** (2.52 – 11.14)	-0.12 * (-0.24 – -0.01)	0.64 (-0.09 – 1.38)
Education	1.07 ** (0.41 – 1.73)	1.55 *** (1.02 – 2.09)	0.04 *** (0.02 – 0.05)	0.07 (-0.02 – 0.16)
Income	0.31 * (0.06 – 0.56)	0.32 ** (0.12 – 0.52)	0.00 (-0.00 – 0.01)	0.02 (-0.02 – 0.05)
Emissions per capita	-0.13 (-0.87 – 0.62)	0.03 (-0.61 – 0.68)	-0.01 (-0.03 – 0.02)	-0.12 ** (-0.20 – -0.03)
GDP per capita	-0.51 (-1.48 – 0.45)	-0.89 * (-1.71 – -0.06)	-0.04 * (-0.07 – -0.01)	0.02 (-0.09 – 0.13)
Dynamic Norm * Tightness	-1.19 (-5.82 – 3.44)	-2.34 (-6.11 – 1.42)	0.01 (-0.09 – 0.11)	-0.07 (-0.71 – 0.57)
Work-together Norm * Tightness	4.41 (-0.26 – 9.08)	2.04 (-1.76 – 5.84)	0.01 (-0.09 – 0.11)	-0.12 (-0.77 – 0.52)
Pluralistic Ignorance * Tightness	-5.75 * (-10.42 – -1.09)	-4.88 * (-8.67 – -1.08)	-0.03 (-0.13 – 0.07)	-0.60 (-1.25 – 0.04)
**Random Effects**				
σ^2	544.05	358.89	0.20	10.45
τ_{00}	31.02 Country	23.02 Country	0.04 Country	0.41 Country
ICC	0.05	0.06	0.15	0.04
N	41 Country	41 Country	41 Country	41 Country
Observations	12760	12722	9794	12769
Marginal R^2 / Conditional R^2	0.024 / 0.077	0.021 / 0.080	0.064 / 0.205	0.043 / 0.079

* *p<0.05* ** *p<0.01* *** *p<0.001*

Main Hypothesized Models With Clustered Standard Errors by Country

The following tables represent the results of the main models reported in the manuscript using an alternative analytical approach. Rather than using multi-level mixed models, we instead ran each regression as a linear regression, using clustered standard errors rather than including a random effect for country. Each outcome is represented in a different table: Belief in Climate Change in Table S9, Climate Mitigation Policy Support in Table S10, Social Media Post in Table S11, and Tree Planting Task in Table S12. For each of the tables, Model 1 represents the model without any covariates. Model 2 represents the model with the individual- and national-level covariates, and Model 3 includes also the national estimates of cultural individualism-collectivism.

Belief in Climate Change

We found similar results as with the mixed-effects model. Specifically, there was a positive interaction effect between cultural tightness and the Work Together Norm condition in Models 2 and 3. There was also a negative interaction with the Pluralistic Ignorance condition in all three models. As in the main manuscript, there was no moderation effect on the Dynamic Norm condition.

Table S9.
Predicting Belief in Climate Change with Clustered Standard Errors by Country.

	Model 1	Model 2	Model 3
Predictors	*Estimates*	*Estimates*	*Estimates*
Intercept	77.27***	75.39***	75.34***
	(72.83 - 81.71)	(71.43 - 79.35)	(71.98 - 78.69)
Cultural Tightness	7.75	9.96	5.21**
	(-1.53 -17.03)	(-0.36 - 20.27)	(-3.03 - 13.45)
Dynamic Norm	0.97	1.04	1.07
	(-0.21 - 2.14)	(-0.43 - 2.50)	(-0.34 - 2.47)
Work-together Norm	-0.29	-0.10	-0.11
	(-1.85 - 1.26)	(-1.88 - 1.68)	(-1.83 - 1.61)
Pluralistic Ignorance	0.77	0.88	0.94
	(-0.28 - 1.81)	(-0.74 - 2.50)	(-0.61 - 2.50)
Dynamic Norm * Tightness	-2.67	-0.25	-0.16
	(-7.41 - 2.07)	(-5.16 - 4.65)	(-5.10 - 4.77)
Work-together Norm * Tightness	2.68	4.69*	4.80*
	(-1.916 - 7.27)	(0.14 - 9.25)	(0.37 - 9.22)
Pluralistic Ignorance * Tightness	-6.08**	-5.51*	-5.44*
	(-9.845 - -2.31)	(-10.36 - -0.66)	(-10.30 - -0.58)
Age		-0.17*	-0.12
		(-0.33 - -0.02)	(-0.29 - 0.04)
Gender: Female		3.55***	3.97***
		(2.38 - 4.73)	(2.61 - 5.32)
Gender: Non-binary/other		3.75	4.49
		(-2.24 - 9.75)	(-1.61 - 10.60)
Education		1.32*	0.55
		(0.11 - 2.53)	(-0.54 - 1.64)
Income		0.11	0.39
		(-0.37 - 0.59)	(-0.12 - 0.89)
Political orientation		-0.22**	-0.23**
		(-0.36 - -0.07)	(-0.37 - -0.10)
Emissions per capita		0.003	0.85
		(-0.82 - 0.82)	(-0.05 - 1.75)
GDP per capita		0.55	0.35
		(-1.01 - 2.10)	(-0.94 - 1.64)
Individualism			-0.21***
			(-0.29 - -0.12)

* *p<0.05* ** *p<0.01* *** *p<0.001*

Climate Mitigation Policy Support

We found similar results as with the mixed-effects model. Specifically, cultural tightness does not moderate the effects of either the Work Together Norm or the Dynamic Norm conditions on climate policy support. However, we found that cultural tightness negatively moderates the effect of the Pluralistic Ignorance condition on policy support.

Table S10.

Predicting Climate Mitigation Policy Support with Clustered Standard Errors by Country.

Predictors	**Model 1** *Estimates*	**Model 2** *Estimates*	**Model 3** *Estimates*
Intercept	68.46*** (65.73 - 71.18)	67.90*** (65.34 - 70.46)	67.85*** (65.92 - 69.79)
Cultural Tightness	5.89 (-1.50 - 13.28)	8.10 (-0.13 - 16.32)	3.83 (-1.93 - 9.58)
Dynamic Norm	1.38** (0.43 - 2.33)	1.31* (0.04 - 2.58)	1.34* (0.14 - 2.53)
Work-together Norm	0.19 (-0.92 - 1.31)	0.25 (-1.06 - 1.56)	0.23 (-1.03 - 1.49)
Pluralistic Ignorance	0.44 (-0.28 - 1.16)	0.69 (-0.27 - 1.65)	0.75 (-0.19 - 1.68)
Dynamic Norm * Tightness	-2.30 (-6.55 - 0.56)	-1.89 (-6.08 - 2.31)	-1.86 (-6.02 - 2.29)
Work-together Norm * Tightness	1.25 (-2.33 - 4.83)	1.96 (-2.54 - 6.47)	2.03 (-2.37 - 6.43)
Pluralistic Ignorance * Tightness	-4.75** (-7.98 - -1.52)	-5.08* (-9.66 - -0.49)	-5.06* (-9.86 - -0.25)
Age		-0.08 (-0.19 - 0.04)	-0.03 (-0.16 - 0.10)
Gender: Female		1.14 (-0.40 - 2.68)	1.52 (-0.18 - 3.22)
Gender: Non-binary/other		3.68 (-0.32 - 7.68)	4.36* (0.17 - 8.54)
Education		2.26*** (1.10 - 3.42)	1.55** (0.58 - 2.53)
Income		0.07 (-0.35 - 0.50)	0.33 (-0.07 - 0.72)
Political orientation		-0.13* (-0.23 - -0.02)	-0.14** (-0.23 - -0.05)
Emissions per capita		0.09 (-0.68 - 0.87)	0.87* (0.08 - 1.65)
GDP per capita		-0.05 (-1.31 - 1.21)	-0.23 (-1.21 - 0.75)
Individualism			-0.19*** (-0.27 - -0.11)

* $p<0.05$ ** $p<0.01$ *** $p<0.001$

Social Media Post Sharing Intention

We found similar results as with the mixed-effects model when using this approach. Specifically, we found no significant interaction effects between cultural tightness and any norm condition on the intention to share climate mitigation information on social media.

Table S11.
Predicting Social Media Post Sharing Intention with Clustered Standard Errors by Country.

Predictors	**Model 1** *Estimates*	**Model 2** *Estimates*	**Model 3** *Estimates*
Intercept	0.48***	0.53***	0.53***
	(0.41 - 0.55)	(0.47 - 0.59)	(0.47 - 0.59)
Cultural Tightness	0.38**	0.30*	0.16
	(0.14 - 0.62)	(0.08 - 0.52)	(-0.04 - 0.36)
Dynamic Norm	0.08***	0.07***	0.07***
	(0.06 - 0.10)	(0.04 - 0.09)	(0.04 - 0.09)
Work-together Norm	0.07***	0.05***	0.05***
	(0.04 - 0.09)	(0.03 - 0.07)	(0.03 - 0.07)
Pluralistic Ignorance	0.02	0.02	0.02
	(-0.005 - 0.05)	(-0.01 - 0.05)	(-0.01 - 0.04)
Dynamic Norm * Tightness	-0.02	-0.001	-0.001
	(-0.09 - 0.06)	(-0.10 - 0.10)	(-0.10 - 0.10)
Work-together Norm * Tightness	-0.05	-0.008	0.001
	(-0.16 - 0.06)	(-0.10 - 0.08)	(-0.09 - 0.09)
Pluralistic Ignorance * Tightness	-0.05	-0.04	-0.04
	(-0.15 - 0.06)	(-0.13 - 0.06)	(-0.13 - 0.06)
Age		-0.002	-0.001
		(-0.007 - 0.002)	(-0.006 - 0.004)
Gender: Female		-0.06*	-0.05
		(-0.10 - -0.01)	(-0.09 - 0.003)
Gender: Non-binary/other		-0.10	-0.07
		(-0.39 - 0.20)	(-0.38 - 0.23)
Education		0.06**	0.04**
		(0.03 - 0.09)	(0.01 - 0.07)
Income		0.004	0.01*
		(-0.01 - 0.02)	(0.0003 - 0.02)
Political orientation		0.002*	0.001*
		(0.0002 - 0.003)	(0.0001 - 0.002)
Emissions per capita		-0.01	0.01
		(-0.04 - 0.01)	(-0.01 - 0.03)
GDP per capita		-0.03	-0.04*
		(-0.06 - 0.005)	(-0.06 - -0.009)
Individualism			-0.006**
			(-0.009 - -0.003)

* *p<0.05* ** *p<0.01* *** *p<0.001*

Tree Planting Task

Similarly to the mixed-effects model, there were no significant moderation effects between cultural tightness and any of the norm conditions on the tree planting task. The only difference is that in the mixed-effect model without covariates (i.e., Model 1) the interaction between cultural tightness and the Pluralistic Ignorance was significant, whereas in this approach it is not. In both cases, the effect is, descriptively, negative. And, across Models 2 and 3 in both cases the effect is negative but non-significant. Using these different approaches may have resulted in slightly different estimates of the effect and different standard errors, leading to this difference in significance for an effect that was on the verge of significant in the main text. Otherwise, the effects remain consistent.

Table S12.
Predicting Tree Planting with Clustered Standard Errors by Country.

	**Model 1**	**Model 2**	**Model 3**
Predictors	*Estimates*	*Estimates*	*Estimates*
Intercept	5.03***	4.76***	4.76***
	(4.77 - 5.29)	(4.41 - 5.11)	(4.44 - 5.07)
Cultural Tightness	-0.23	-0.01	-0.38
	(-1.32 - 0.86)	(-1.22 - 1.20)	(-1.47 - 0.72)
Dynamic Norm	0.008	-0.03	-0.03
	(-0.16 - 0.18)	(-0.23 - 0.17)	(-0.23 - 0.18)
Work-together Norm	-0.40***	-0.42***	-0.42***
	(-0.54 - -0.25)	(-0.59 - -0.25)	(-0.59 - -0.26)
Pluralistic Ignorance	-0.14	-0.14	-0.13
	(-0.32 - 0.05)	(-0.33 - 0.054)	(-0.32 - 0.06)
Dynamic Norm * Tightness	-0.26	0.20	0.21
	(-0.97 - 0.46)	(-0.52 - 0.93)	(-0.51 - 0.93)
Work-together Norm * Tightness	0.04	0.17	0.18
	(-0.50 - 0.59)	(-0.37 - 0.71)	(-0.34 - 0.70)
Pluralistic Ignorance * Tightness	-0.48	-0.50	-0.49
	(-1.25 - 0.28)	(-1.20 - 0.21)	(-1.19 - 0.20)
Age		0.04***	0.05
		(0.03 - 0.05)	(0.04 - 0.06)
Gender: Female		0.63***	0.66
		(0.41 - 0.85)	(0.45 - 0.88)
Gender: Non-binary/other		0.39	0.45
		(-0.57 - 1.35)	(-0.50 - 1.40)
Education		0.13	0.07
		(-0.03 - 0.29)	(-0.06 - 0.20)
Income		-0.01	0.01
		(-0.07 - 0.05)	(-0.04 - 0.06)
Political orientation		-0.005*	-0.007**
		(-0.01 - -0.0004)	(-0.01 - -0.002)
Emissions per capita		-0.14**	-0.08
		(-0.23 - -0.05)	(-0.18 - 0.03)
GDP per capita		0.07	0.05
		(-0.06 - 0.19)	(-0.05 - 0.16)
Individualism			-0.02**
			(-0.03 - -0.006)

* *p<0.05* ** *p<0.01* *** *p<0.001*

Comparisons Between Experimental Conditions

In addition to the comparisons between each experimental condition and the Control condition, we tested the differences between experimental conditions in their interactions with cultural tightness, to see if there is evidence that some conditions are significantly more affected by cultural tightness than others.

In predicting Belief in Climate Change, the comparisons between the Dynamic Norms condition and both the Work-Together Norm (b = 5.12, 95% CI [0.58 - 9.66], SE = 2.32, p = .03) and the Pluralistic Ignorance conditions (b = -5.18, 95% CI [-9.71 - -0.65], SE = 2.31, p = .03) were significant. Additionally, the interaction comparing between the Work-Together Norm condition and the Pluralistic Ignorance condition was significant (b = -10.30, 95% CI [-14.87 - -5.73], SE = 2.33, p < .001). The Work-Together Norm condition was significantly more positive in its relationship with cultural tightness and belief than any other condition. Conversely, the Pluralistic Ignorance condition was significantly more negative than all other conditions.

For Policy Support, we see a similar pattern. The interaction comparing the Dynamic Norms and Work-Together Norm conditions (b = -4.11, 95% CI [0.38 - 7.83], SE = 1.90, p < .05) is significant, as is the comparison between the Work-Together Norm and Pluralistic Ignorance conditions (b = -7.04, 95% CI [-10.79 - -3.29], SE = 1.91, p < .001). In both cases, the Work-Together Norm condition's relationship between cultural tightness and policy support was more positive than the other conditions. The interaction comparing the Dynamic Norm condition and the Pluralistic Ignorance condition was not significant however (b = -2.93, 95% CI [-6.65 - 0.79], SE = 1.90, p = .12).

For the model predicting Social Media Post Sharing, we find no differences in the slopes comparing any of the three experimental conditions (ps > .39).

Finally, for the Tree Planting Task, the interaction comparing the Dynamic Norm condition and the Work-Together Norm conditions was not significant (*b* = -0.14, 95% CI [-0.75 - 0.46], *SE* = 0.31, *p* = .65). However, the interaction of the cultural tightness with the comparison between Dynamic Norm condition and the Pluralistic Ignorance condition was significant (*b* = -0.61, 95% CI [-1.21 - -0.001], *SE* = 0.31, *p* = .049), but it was not the interaction with the comparison between the Work-Together Norm condition and the Pluralistic Ignorance condition (*b* = -0.47, 95% CI [-1.08 - 0.15], *SE* = 0.31, *p* = .13). Thus, the Pluralistic Ignorance condition had a significantly more negative relationship between cultural tightness and the Tree Planting Task than the Dynamic Norm condition.

Pluralistic Ignorance Supplemental Analyses

The following tables represent the results of the exploratory models only comparing the Control and the Pluralistic Ignorance condition. In each table are reported all the four outcomes (Belief in Climate Change, Policy Support, Social Media Post, Tree Planting). Table S13 represents the model reported in the main text, accounting for participants' prior estimates of the norm as a covariate. For this analysis, participants' prior estimates were accounted for with a covariate that was the difference between their estimate and their nation's actual norm estimate. This measure, the Pluralistic Ignorance ΔEstimate was computed as follows:

$$PI \otimes Estimate = PI_{subj.} - PI_{real},$$

where $PI_{subj.}$ represents the percentage participants reported, and PI_{real} represents the real percentage reported in the UNDP (2021) report.

Additionally, given that the Pluralistic Ignorance intervention targets our outcomes by correcting misperceptions, it is possible that under- versus over-estimating the norm might yield different effects on our four outcomes. Thus Table S14 reports the results of a three-way interaction between cultural tightness, the Pluralistic Ignorance intervention compared to the Control, and a dummy-coded variable indicating whether the participant under- or over-estimated the norm.

Another way to account for variance in the Pluralistic Ignorance condition is to simply control for the norm percentage displayed to participants in the intervention, rather than the difference between a participant's estimate and the actual percentage. Because different countries had different norm reference points, controlling for this factor can help examine the effects of the Pluralistic Ignorance intervention across all levels of norm reference points used. Table S15 thus represents the model in the main text, additionally controlling for the percentage in the participant's country.

Finally, because some countries used a norm estimate of their specific country in the Pluralistic Ignorance condition, while others used a regional estimate, we accounted for this as a binary control

variable in a supplemental model. Table S16 represents the model reported in the main text with also the Pluralistic Ignorance geographic area (country vs. region) reported in the manipulation as a covariate.

Pluralistic Ignorance ⊗Estimate as covariate

For all four outcomes, the Pluralistic Ignorance ⊗Estimate was a significant positive predictor. Specifically, the more people overestimate the actual percentage of people in their country believing that "Climate change is a global emergency", the more people reported higher belief in climate change, higher climate mitigation policy support, higher intention to share climate information on social media and more tree planting in the behavioral task.

Table S13.

Interactive Effects of Cultural Tightness, Pluralistic Norm Intervention, and Prior Norm Estimates on Climate Change Attitudes and Behaviors.

	Belief in Climate Change	Policy Support	Social Media Post	Tree Planting
Predictors	*Estimates*	*Estimates*	*Estimates*	*Estimates*
Intercept	79.78 *** (77.86 – 81.69)	71.50 *** (69.85 – 73.15)	0.56 *** (0.50 – 0.62)	4.26 *** (4.00 – 4.53)
Cultural Tightness	3.82 (-2.59 – 10.22)	5.77 * (0.24 – 11.30)	0.23 * (0.03 – 0.42)	0.04 (-0.84 – 0.93)
Pluralistic Ignorance	0.64 (-0.41 – 1.68)	0.46 (-0.40 – 1.33)	0.02 (-0.01 – 0.04)	-0.11 (-0.27 – 0.05)
ΔEstimate	0.40 *** (0.38 – 0.43)	0.29 *** (0.27 – 0.31)	0.00 *** (0.00 – 0.01)	0.00 * (0.00 – 0.01)
Age	-0.07 *** (-0.11 – -0.03)	0.02 (-0.01 – 0.05)	-0.00 *** (-0.00 – -0.00)	0.04 *** (0.04 – 0.05)
Gender: Female	3.22 *** (2.15 – 4.29)	0.70 (-0.19 – 1.60)	-0.06 *** (-0.09 – -0.03)	0.47 *** (0.31 – 0.63)
Gender: Non-binary/other	1.88 (-4.84 – 8.61)	3.81 (-1.78 – 9.41)	-0.09 (-0.25 – 0.07)	0.68 (-0.35 – 1.70)
Education	1.50 *** (0.66 – 2.34)	1.70 *** (1.00 – 2.40)	0.04 *** (0.02 – 0.06)	0.01 (-0.12 – 0.13)
Income	0.09 (-0.22 – 0.41)	0.27 * (0.00 – 0.53)	0.00 (-0.01 – 0.01)	0.04 (-0.01 – 0.09)
Political orientation	-0.25 *** (-0.27 – -0.23)	-0.16 *** (-0.18 – -0.14)	0.00 (-0.00 – 0.00)	-0.01 *** (-0.01 – -0.00)
Emissions per capita	-0.03 (-0.73 – 0.67)	0.22 (-0.38 – 0.83)	-0.01 (-0.03 – 0.02)	-0.13 ** (-0.23 – -0.04)
GDP per capita	0.11 (-0.79 – 1.00)	-0.43 (-1.21 – 0.35)	-0.04 * (-0.06 – -0.01)	0.03 (-0.09 – 0.16)
Pluralistic Ignorance * Tightness	-6.29 ** (-10.52 – -2.06)	-5.26 ** (-8.78 – -1.74)	-0.05 (-0.15 – 0.05)	-0.60 (-1.24 – 0.05)
**Random Effects**				
σ^2	444.51	307.58	0.20	10.35
τ_{00}	25.18 Country	19.22 Country	0.03 Country	0.45 Country
ICC	0.05	0.06	0.12	0.04
N	41 Country	41 Country	41 Country	41 Country
Observations	6353	6339	4877	6357
Marginal R^2 / Conditional R^2	0.191 / 0.234	0.145 / 0.195	0.107 / 0.215	0.050 / 0.089

* *p<0.05* ** *p<0.01* *** *p<0.001*

Pluralistic Ignorance Under- vs. Overestimation of the Norm

We find significant interactions between cultural tightness and whether someone over- or underestimates their national/regional norm for both belief in climate change and willingness to share a social media post. However, norm estimation did not significantly interact with either the condition variable directly or in the three-way interaction for any outcome variable. So, comparisons between the Control and Pluralistic Ignorance conditions did not change between over- and underestimate of their national norms.

Table S14.

Interactive Effects of Cultural Tightness, Pluralistic Norm Intervention, and Under- vs. Overestimation of the norm on Climate Change Attitudes and Behaviors.

	**Belief in Climate Change**	**Policy Support**	**Social Media Post**	**Tree Planting**
Predictors	*Estimates*	*Estimates*	*Estimates*	*Estimates*
Intercept	85.32 *** (83.18 – 87.46)	75.37 *** (73.54 – 77.21)	0.65 *** (0.58 – 0.71)	4.26 *** (3.96 – 4.56)
Pluralistic Ignorance	0.04 (-1.65 – 1.72)	-0.32 (-1.72 – 1.07)	-0.01 (-0.05 – 0.03)	-0.10 (-0.35 – 0.15)
Cultural Tightness	0.50 (-6.66 – 7.66)	5.37 (-0.79 – 11.52)	0.11 (-0.11 – 0.33)	0.20 (-0.80 – 1.21)
Estimate: Underestimate	-13.42 *** (-15.05 – -11.79)	-9.59 *** (-10.94 – -8.24)	-0.19 *** (-0.23 – -0.15)	-0.05 (-0.29 – 0.19)
Age	-0.08 *** (-0.12 – -0.04)	0.02 (-0.02 – 0.05)	-0.00 *** (-0.00 – -0.00)	0.04 *** (0.03 – 0.05)
Gender: Female	3.56 *** (2.44 – 4.67)	0.98 * (0.06 – 1.90)	-0.06 *** (-0.08 – -0.03)	0.47 *** (0.31 – 0.64)
Gender: Non-binary/other	1.38 (-5.60 – 8.37)	3.53 (-2.24 – 9.31)	-0.09 (-0.25 – 0.07)	0.68 (-0.34 – 1.71)
Education	1.64 *** (0.77 – 2.51)	1.80 *** (1.08 – 2.52)	0.05 *** (0.03 – 0.07)	0.01 (-0.12 – 0.13)
Income	0.13 (-0.20 – 0.45)	0.30 * (0.03 – 0.57)	0.00 (-0.01 – 0.01)	0.04 (-0.01 – 0.09)
Political orientation	-0.24 *** (-0.26 – -0.21)	-0.15 *** (-0.17 – -0.13)	0.00 (-0.00 – 0.00)	-0.01 *** (-0.01 – -0.00)
Emissions per capita	-0.09 (-0.78 – 0.60)	0.17 (-0.44 – 0.77)	-0.01 (-0.03 – 0.02)	-0.13 ** (-0.23 – -0.04)
GDP per capita	-0.07 (-0.96 – 0.82)	-0.58 (-1.36 – 0.20)	-0.04 * (-0.07 – -0.01)	0.03 (-0.09 – 0.15)
Pluralistic Ignorance * Tightness	-3.64 (-9.83 – 2.55)	-5.44 * (-10.56 – -0.32)	0.03 (-0.11 – 0.17)	-0.70 (-1.61 – 0.21)
Pluralistic Ignorance * Estimate: Underestimate	1.03 (-1.18 – 3.24)	1.50 (-0.33 – 3.33)	0.04 (-0.02 – 0.09)	-0.01 (-0.33 – 0.32)
Tightness * Estimate: Underestimate	7.20 * (0.84 – 13.57)	1.26 (-4.01 – 6.52)	0.23 ** (0.09 – 0.37)	-0.28 (-1.22 – 0.65)
Pluralistic Ignorance * Tightness * Estimate: Underestimate	-2.48 (-11.34 – 6.38)	2.79 (-4.54 – 10.12)	-0.11 (-0.31 – 0.09)	0.20 (-1.10 – 1.51)
**Random Effects**				
σ^2	478.68	327.52	0.20	10.36
τ_{00}	24.35 Country	18.90 Country	0.03 Country	0.45 Country
ICC	0.05	0.05	0.13	0.04
N	41 Country	41 Country	41 Country	41 Country

Observations	6353	6339	4877	6357
Marginal R^2 / Conditional R^2	0.126 / 0.169	0.089 / 0.138	0.097 / 0.215	0.049 / 0.089

* $p<0.05$ ** $p<0.01$ *** $p<0.001$

Pluralistic Ignorance Norm Percentage

We find that including the norm percentage displayed to participants in the Pluralistic Ignorance condition is a significant predictor in our model predicting willingness to post on social media; however, this effect is small. The inclusion of the norm percentage as a covariate does not change the results of the Pluralistic Ignorance experimental condition and is not a significant predictor in the other three models.

Table S15.

Interactive Effects of Cultural Tightness, Pluralistic Norm Intervention, and Actual Norm Percentages on Climate Change Attitudes and Behaviors.

	**Belief in Climate Change**	**Policy Support**	**Social Media Post**	**Tree Planting**
Predictors	*Estimates*	*Estimates*	*Estimates*	*Estimates*
Intercept	83.65 *** (61.76 – 105.54)	84.86 *** (66.73 – 102.98)	1.28 *** (0.58 – 1.97)	4.57 ** (1.82 – 7.32)
Pluralistic Ignorance	0.94 (-0.18 – 2.06)	0.69 (-0.23 – 1.60)	0.02 (-0.01 – 0.04)	-0.11 (-0.27 – 0.05)
Cultural Tightness	6.42 (-0.67 – 13.51)	7.18 * (1.33 – 13.04)	0.23 * (0.01 – 0.44)	0.06 (-0.85 – 0.96)
Norm Percentage	-0.09 (-0.42 – 0.23)	-0.23 (-0.50 – 0.04)	-0.01 * (-0.02 – -0.00)	-0.01 (-0.05 – 0.04)
Age	-0.08 *** (-0.12 – -0.05)	0.01 (-0.02 – 0.05)	-0.00 *** (-0.00 – -0.00)	0.04 *** (0.03 – 0.05)
Gender: Female	3.93 *** (2.78 – 5.08)	1.22 * (0.28 – 2.17)	-0.05 *** (-0.08 – -0.03)	0.48 *** (0.31 – 0.64)
Gender: Non-binary/other	2.40 (-4.84 – 9.63)	4.19 (-1.73 – 10.11)	-0.09 (-0.25 – 0.07)	0.68 (-0.34 – 1.71)
Education	1.59 *** (0.69 – 2.49)	1.75 *** (1.01 – 2.49)	0.05 *** (0.02 – 0.07)	0.01 (-0.12 – 0.13)
Income	0.29 (-0.05 – 0.63)	0.41 ** (0.14 – 0.69)	0.00 (-0.00 – 0.01)	0.04 (-0.01 – 0.09)
Political orientation	-0.21 *** (-0.23 – -0.18)	-0.13 *** (-0.15 – -0.11)	0.00 ** (0.00 – 0.00)	-0.01 *** (-0.01 – -0.00)
Emissions per capita	-0.23 (-1.03 – 0.57)	0.18 (-0.48 – 0.84)	-0.00 (-0.03 – 0.02)	-0.13 * (-0.23 – -0.03)
GDP per capita	-0.37 (-1.38 – 0.65)	-0.66 (-1.50 – 0.18)	-0.03 * (-0.07 – -0.00)	0.03 (-0.09 – 0.16)
Pluralistic Ignorance * Tightness	-5.53 * (-10.08 – -0.99)	-4.72 * (-8.44 – -1.00)	-0.04 (-0.14 – 0.06)	-0.59 (-1.24 – 0.05)
**Random Effects**				
σ^2	513.98	344.03	0.20	10.35
τ_{00}	30.61 Country	21.04 Country	0.03 Country	0.47 Country
ICC	0.06	0.06	0.14	0.04
N	41 Country	41 Country	41 Country	41 Country
Observations	6353	6339	4877	6357
Marginal R^2 / Conditional R^2	0.061 / 0.114	0.044 / 0.099	0.085 / 0.212	0.049 / 0.090

* *p<0.05* ** *p<0.01* *** *p<0.001*

Pluralistic Ignorance PI Area (Region vs. Country)

For none of the four outcomes the Pluralistic Ignorance Area of the actual estimates (UNDP, 2021) reached the statistical significance.

Table S16.

Predicting Climate Change Attitudes and Behavior Controlling for Pluralistic Ignorance Reference (Region vs. Country).

	**Belief in Climate Change**	**Policy Support**	**Social Media Post**	**Tree Planting**
Predictors	*Estimates*	*Estimates*	*Estimates*	*Estimates*
Intercept	76.42 ***	69.31 ***	0.52 ***	4.26 ***
	(73.65 – 79.19)	(66.94 – 71.69)	(0.43 – 0.61)	(3.90 – 4.62)
Cultural Tightness	6.48	7.77 *	0.26 *	0.08
	(-0.48 – 13.43)	(1.80 – 13.73)	(0.03 – 0.48)	(-0.82 – 0.98)
Pluralistic Ignorance	0.93	0.68	0.02	-0.11
	(-0.19 – 2.05)	(-0.24 – 1.60)	(-0.01 – 0.04)	(-0.27 – 0.05)
Area	1.99	0.96	0.02	-0.04
	(-1.68 – 5.66)	(-2.20 – 4.13)	(-0.10 – 0.15)	(-0.51 – 0.42)
Age	-0.09 ***	0.01	-0.00 ***	0.04 ***
	(-0.13 – -0.05)	(-0.02 – 0.05)	(-0.00 – -0.00)	(0.03 – 0.05)
Gender: Female	3.93 ***	1.22 *	-0.05 ***	0.48 ***
	(2.78 – 5.08)	(0.28 – 2.16)	(-0.08 – -0.03)	(0.31 – 0.64)
Gender: Non-binary/other	2.38<br>(-4.86 – 9.61)	4.18<br>(-1.74 – 10.10)	-0.09<br>(-0.25 – 0.07)	0.68<br>(-0.34 – 1.71)
Education	1.60 ***	1.76 ***	0.05 ***	0.01
	(0.70 – 2.50)	(1.02 – 2.50)	(0.02 – 0.07)	(-0.12 – 0.13)
Income	0.30	0.41 **	0.00	0.04
	(-0.04 – 0.63)	(0.14 – 0.69)	(-0.00 – 0.01)	(-0.01 – 0.09)
Political orientation	-0.21 ***	-0.13 ***	0.00 **	-0.01 ***
	(-0.23 – -0.18)	(-0.15 – -0.11)	(0.00 – 0.00)	(-0.01 – -0.00)
Emissions per capita	-0.24	0.05	-0.01	-0.14 **
	(-1.01 – 0.52)	(-0.60 – 0.71)	(-0.03 – 0.02)	(-0.23 – -0.04)
GDP per capita	-0.43	-0.83	-0.04 *	0.03
	(-1.41 – 0.54)	(-1.67 – 0.01)	(-0.08 – -0.01)	(-0.09 – 0.15)
Pluralistic Ignorance * Tightness	-5.54 *<br>(-10.09 – -1.00)	-4.72 *<br>(-8.44 – -1.00)	-0.04<br>(-0.14 – 0.06)	-0.59<br>(-1.24 – 0.05)
**Random Effects**				
σ^2	513.98	344.03	0.20	10.35
τ_{00}	29.75 Country	22.52 Country	0.04 Country	0.46 Country
ICC	0.05	0.06	0.15	0.04
N	41 Country	41 Country	41 Country	41 Country
Observations	6353	6339	4877	6357
Marginal R^2 / Conditional R^2	0.063 / 0.114	0.044 / 0.103	0.067 / 0.210	0.049 / 0.090

* *p<0.05* ** *p<0.01* *** *p<0.001*

Models with an Alternative Cultural Tightness Measure (Uz, 2015)

The following tables represent the results of the models reported in the main text but with a different measure of cultural tightness collected by Uz (2015). By using the Uz dataset for cultural tightness, we are able to add the following countries to our analysis: Belgium, Bulgaria, Denmark, France, Gambia, Morocco, Norway, Philippines, Romania, Serbia, Slovenia, Sudan, Switzerland, Tanzania, Uganda, Uruguay. However, compared to the Eriksson et al. dataset, we simultaneously lose the following countries: Czech Republic, Saudi Arabia, South Korea, Sri Lanka, United Arab Emirates, United Kingdom, United States.

Each outcome is represented in a different table: Belief in Climate Change in Table S11, Climate Mitigation Policy Support in Table S12, Social Media Post in Table S13, Tree Planting Task in Table S14. For each of the tables, Model 1 represents the model without any covariates. Model 2 represents the model with the individual- and national-level covariates, and Model 3 includes also the national estimates of cultural individualism-collectivism.

Belief in Climate Change

In all three models, none of the three interactions reached statistical significance.

Table S17.
Predicting Belief in Climate Change With an Alternative Measure of Cultural Tightness.

Predictors	**Model 1** *Estimates*	**Model 2** *Estimates*	**Model 3** *Estimates*
Intercept	80.69 ***	78.51 ***	77.71 ***
	(78.40 – 82.97)	(76.33 – 80.70)	(75.99 – 79.42)
Cultural Tightness	0.05	0.01	-0.12 **
	(-0.04 – 0.13)	(-0.08 – 0.09)	(-0.20 – -0.04)
Dynamic Norm	0.59	0.07	0.08
	(-0.55 – 1.73)	(-1.23 – 1.36)	(-1.22 – 1.37)
Work-together Norm	-1.90 **	-1.94 **	-1.94 **
	(-3.05 – -0.76)	(-3.24 – -0.64)	(-3.24 – -0.64)
Pluralistic Ignorance	0.31	-0.29	-0.28
	(-0.83 – 1.44)	(-1.59 – 1.00)	(-1.57 – 1.01)
Dynamic Norm * Tightness	0.02	0.03	0.03
	(-0.02 – 0.07)	(-0.02 – 0.08)	(-0.02 – 0.08)
Work-together Norm * Tightness	0.04	0.03	0.03
	(-0.00 – 0.09)	(-0.02 – 0.08)	(-0.02 – 0.08)
Pluralistic Ignorance * Tightness	-0.01	-0.01	-0.01
	(-0.05 – 0.04)	(-0.06 – 0.04)	(-0.06 – 0.04)
Age		-0.03 *	-0.03 *
		(-0.07 – -0.00)	(-0.07 – -0.00)
Gender: Female		5.09 ***	5.10 ***
		(4.15 – 6.02)	(4.17 – 6.04)
Gender: Non-binary/other		4.12	4.19
		(-2.24 – 10.48)	(-2.17 – 10.54)
Education		0.70	0.67
		(-0.02 – 1.42)	(-0.05 – 1.39)
Income		0.26	0.25
		(-0.02 – 0.54)	(-0.03 – 0.53)
Political orientation		-0.15 ***	-0.15 ***
		(-0.17 – -0.13)	(-0.18 – -0.13)
Emissions per capita		-0.58	-0.02
		(-1.36 – 0.21)	(-0.61 – 0.58)
GDP per capita		-0.71	-0.07
		(-1.70 – 0.27)	(-0.81 – 0.67)
Individualism			-2.50 ***
			(-3.40 – -1.61)
**Random Effects**			
σ^2	485.64	461.02	460.98
τ_{00}	41.29 Country	30.21 Country	14.40 Country
ICC	0.08	0.06	0.03
N	37 Country	35 Country	35 Country
Observations	11567	8569	8569
Marginal R^2 / Conditional R^2	0.007 / 0.084	0.052 / 0.110	0.076 / 0.104

** p<0.05 ** p<0.01 *** p<0.001*

Climate Mitigation Policy Support

Across all three models, only the Pluralistic Ignorance condition significantly interacts with cultural tightness. Specifically, this interaction is significantly negative, consistent with our findings using the Erikson et al. (2021) measure of cultural tightness.

Table S18.

Predicting Climate Mitigation Policy Support With an Alternative Measure of Cultural Tightness.

Predictors	**Model 1** *Estimates*	**Model 2** *Estimates*	**Model 3** *Estimates*
Intercept	70.16 *** (68.28 – 72.03)	69.25 *** (67.29 – 71.20)	68.66 *** (66.93 – 70.38)
Cultural Tightness	0.08 * (0.01 – 0.15)	0.07 (-0.01 – 0.15)	-0.02 (-0.10 – 0.06)
Dynamic Norm	1.36 ** (0.44 – 2.28)	0.91 (-0.14 – 1.97)	0.92 (-0.13 – 1.97)
Work-together Norm	-0.48 (-1.40 – 0.45)	-0.66 (-1.72 – 0.40)	-0.66 (-1.71 – 0.40)
Pluralistic Ignorance	0.52 (-0.40 – 1.43)	0.41 (-0.64 – 1.46)	0.42 (-0.63 – 1.48)
Dynamic Norm * Tightness	0.02 (-0.02 – 0.05)	0.01 (-0.03 – 0.05)	0.01 (-0.03 – 0.05)
Work-together Norm * Tightness	0.02 (-0.02 – 0.06)	0.03 (-0.01 – 0.07)	0.03 (-0.01 – 0.07)
Pluralistic Ignorance * Tightness	-0.04 * (-0.08 – -0.01)	-0.04 (-0.08 – 0.00)	-0.04 (-0.08 – 0.00)
Age		0.06 *** (0.03 – 0.09)	0.06 *** (0.03 – 0.09)
Gender: Female		2.57 *** (1.80 – 3.33)	2.58 *** (1.81 – 3.34)
Gender: Non-binary/other		3.28 (-1.95 – 8.51)	3.33 (-1.90 – 8.56)
Education		1.09 *** (0.50 – 1.68)	1.09 *** (0.50 – 1.68)
Income		0.25 * (0.02 – 0.48)	0.24 * (0.01 – 0.47)
Political orientation		-0.10 *** (-0.11 – -0.08)	-0.10 *** (-0.11 – -0.08)
Emissions per capita		0.04 (-0.68 – 0.75)	0.44 (-0.20 – 1.08)
GDP per capita		-0.86 (-1.76 – 0.05)	-0.39 (-1.19 – 0.41)
Individualism			-1.82 *** (-2.78 – -0.86)
**Random Effects**			
σ^2	314.36	304.80	304.79
τ_{00}	27.95 Country	25.71 Country	17.80 Country
ICC	0.08	0.08	0.06
N	37 Country	35 Country	35 Country
Observations	11490	8541	8541
Marginal R^2 / Conditional R^2	0.014 / 0.094	0.039 / 0.114	0.055 / 0.108

* *p<0.05* ** *p<0.01* *** *p<0.001*

Social Media Post Sharing Intention

In all three models, none of the three interactions reached statistical significance.

Table S19.

Predicting Social Media Sharing Intentions With an Alternative Measure of Cultural Tightness.

Predictors	**Model 1** *Estimates*	**Model 2** *Estimates*	**Model 3** *Estimates*
Intercept	0.47 ***	0.50 ***	0.47 ***
	(0.39 – 0.54)	(0.42 – 0.58)	(0.41 – 0.54)
Cultural Tightness	0.00	0.00	-0.00
	(-0.00 – 0.01)	(-0.00 – 0.00)	(-0.01 – 0.00)
Dynamic Norm	0.05 ***	0.05 **	0.05 **
	(0.03 – 0.08)	(0.02 – 0.08)	(0.02 – 0.08)
Work-together Norm	0.03 *	0.03	0.03
	(0.01 – 0.06)	(-0.00 – 0.06)	(-0.00 – 0.06)
Pluralistic Ignorance	-0.00	-0.01	-0.01
	(-0.03 – 0.02)	(-0.04 – 0.02)	(-0.04 – 0.02)
Dynamic Norm * Tightness	-0.00	-0.00	-0.00
	(-0.00 – 0.00)	(-0.00 – 0.00)	(-0.00 – 0.00)
Work-together Norm * Tightness	0.00	0.00	0.00
	(-0.00 – 0.00)	(-0.00 – 0.00)	(-0.00 – 0.00)
Pluralistic Ignorance * Tightness	0.00	0.00	0.00
	(-0.00 – 0.00)	(-0.00 – 0.00)	(-0.00 – 0.00)
Age		0.00	0.00
		(-0.00 – 0.00)	(-0.00 – 0.00)
Gender: Female		-0.02	-0.02
		(-0.04 – 0.00)	(-0.04 – 0.00)
Gender: Non-binary/other		0.04	0.04
		(-0.11 – 0.19)	(-0.11 – 0.19)
Education		0.04 ***	0.04 ***
		(0.02 – 0.06)	(0.02 – 0.06)
Income		-0.01	-0.01
		(-0.01 – 0.00)	(-0.01 – 0.00)
Political orientation		0.00 ***	0.00 ***
		(0.00 – 0.00)	(0.00 – 0.00)
Emissions per capita		0.01	0.02
		(-0.02 – 0.04)	(-0.00 – 0.05)
GDP per capita		-0.03	-0.01
		(-0.06 – 0.01)	(-0.04 – 0.03)
Individualism			-0.08 ***
			(-0.11 – -0.04)
**Random Effects**			
σ^2	0.20	0.20	0.20
τ_{00}	0.05 Country	0.04 Country	0.03 Country
ICC	0.20	0.18	0.13
N	37 Country	35 Country	35 Country
Observations	8642	6533	6533
Marginal R^2 / Conditional R^2	0.023 / 0.221	0.033 / 0.211	0.097 / 0.216

* $p<0.05$ ** $p<0.01$ *** $p<0.001$

Tree Planting Task

In all three models, none of the three interactions reached statistical significance.

Table S20.

Predicting the Tree Planting Task With an Alternative Measure of Cultural Tightness.

	**Model 1**	**Model 2**	**Model 3**
Predictors	*Estimates*	*Estimates*	*Estimates*
Intercept	4.14 ***	4.01 ***	3.97 ***
	(3.87 – 4.41)	(3.73 – 4.28)	(3.70 – 4.24)
Cultural Tightness	0.00	0.01	0.00
	(-0.01 – 0.01)	(-0.00 – 0.02)	(-0.01 – 0.01)
Dynamic Norm	0.03	0.01	0.01
	(-0.14 – 0.20)	(-0.18 – 0.21)	(-0.18 – 0.21)
Work-together Norm	-0.33 ***	-0.28 **	-0.28 **
	(-0.50 – -0.16)	(-0.47 – -0.09)	(-0.47 – -0.08)
Pluralistic Ignorance	-0.08	-0.06	-0.06
	(-0.25 – 0.09)	(-0.26 – 0.13)	(-0.25 – 0.13)
Dynamic Norm * Tightness	-0.01	-0.01	-0.01
	(-0.01 – 0.00)	(-0.01 – 0.00)	(-0.01 – 0.00)
Work-together Norm * Tightness	-0.00	-0.00	-0.00
	(-0.01 – 0.00)	(-0.01 – 0.00)	(-0.01 – 0.00)
Pluralistic Ignorance * Tightness	-0.01	-0.01	-0.01
	(-0.01 – 0.00)	(-0.01 – 0.00)	(-0.01 – 0.00)
Age		0.05 ***	0.05 ***
		(0.04 – 0.05)	(0.04 – 0.05)
Gender: Female		0.60 ***	0.60 ***
		(0.46 – 0.74)	(0.46 – 0.74)
Gender: Non-binary/other		0.55	0.55
		(-0.40 – 1.50)	(-0.39 – 1.50)
Education		0.01	0.01
		(-0.10 – 0.12)	(-0.10 – 0.12)
Income		0.03	0.03
		(-0.01 – 0.08)	(-0.01 – 0.08)
Political orientation		-0.01 ***	-0.01 ***
		(-0.01 – -0.01)	(-0.01 – -0.01)
Emissions per capita		-0.11 *	-0.09
		(-0.21 – -0.02)	(-0.18 – 0.01)
GDP per capita		-0.06	-0.03
		(-0.18 – 0.05)	(-0.15 – 0.09)
Individualism			-0.12
			(-0.27 – 0.02)
**Random Effects**			
σ^2	10.69	10.21	10.21
τ_{00}	0.53 Country	0.40 Country	0.38 Country
ICC	0.05	0.04	0.04
N	37 Country	35 Country	35 Country
Observations	11576	8573	8573
Marginal R^2 / Conditional R^2	0.002 / 0.050	0.059 / 0.095	0.059 / 0.092

* $p<0.05$ ** $p<0.01$ *** $p<0.001$

Bayesian Analysis Testing for Support of the Null Hypothesis

Each model testing the interaction of cultural tightness and the social norm interventions reported in the main text was tested using Bayesian multilevel regression analysis (nesting data within countries) to see if there was evidence in support of the null hypothesis. For each model, we tested the evidence ratio in support of the null hypothesis, that the interaction between tightness and each social norm intervention was zero. Models were tested with 5,000 iterations each, including 1,000 warm-ups, using 4 chains. Model fit was evaluated with a comparison between the observed and predicted value plots as well as sufficient minimum and tail effective sample sizes and sufficiently small r-hat values (<1.01). All models met these criteria and resulted in no divergences. These models required the use of non-normal distributions, so the results may not directly translate to the results observed in the main text, which were analyzed using linear regression assumptions. Specifically, we used a zero-one-inflated beta distribution for climate change belief, climate policy support and tree-planting task, and a Bernoulli distribution for intention to share climate mitigation information on social media. Results are reported in Table 3 in the main text.

From these results, we can conclude that we generally have strong evidence in support of the null hypothesis for most outcomes and conditions. However, there is only moderate evidence in support of the null for the Social Media Post results, and for the interaction of cultural tightness and the Pluralistic Ignorance condition on Policy Support (an interaction that was significant in the main text). For all other results, there is strong evidence in support of the null, even for some interactions that were significant in the main text.

Experimental Effects in the U.S.

Whether controlling for covariates or not, we saw some significant, positive effects of the social norm interventions across outcomes. The Tree Planting Task did not systematically differ from the Control in any condition, with only a trending reduction in effort in the task in the Work-Together Norm condition compared to Control. However, we saw that all conditions were higher than the Control in Social Media Post Intentions. The Dynamic Norm condition was higher than Control in Policy Support, and there was some trending evidence that the Pluralistic Ignorance condition was too. The Pluralistic Ignorance condition was also higher in Belief in Climate Change, with the Dynamic Norm being slightly higher than Control as well (though this was only a small trend).

Overall, there were some positive effects of each condition on our outcomes. No condition was consistently significantly higher on all outcomes compared to the Control, due to the largely null effects of the Tree Planting Task. However all were in the expected direction descriptively and many showed significant comparisons to the Control condition.

Table S21.
Experimental Intervention Effects in the U.S.

	Belief in Climate Change		Policy Support		Social Media Post		Tree Planting	
	**Model 1**	**Model 2**	**Model 1**	**Model 2**	**Model 1**	**Model 2**	**Model 1**	**Model 2**
	Estimates	*Estimates*	*Estimates*	*Estimates*	*Estimates*	*Estimates*	*Estimates*	*Estimates*
Dynamic Norm	2.23 (1.62)	2.91† (1.56)	2.64* (1.29)	3.54** (1.29)	0.06* (0.03)	0.07* (0.03)	0.18 (0.18)	0.16 (0.19)
Work-Together Norm	2.47 (1.63)	2.20 (1.57)	1.82 (1.30)	1.87 (1.30)	0.07* (0.03)	0.06† (0.03)	-0.25 (0.18)	-0.33† (0.19)
Pluralistic Ignorance	3.39* (1.63)	3.73* (1.56)	1.33 (1.29)	2.27† (1.29)	0.05† (0.03)	0.06* (0.03)	-0.14 (0.18)	-0.19 (0.19)
Age		-0.28 (0.03)***		-0.17*** (0.03)		-0.01*** (0.00)		0.04*** (0.00)
Gender: Female		3.63 (1.11)**		-0.42 (0.92)		-0.09*** (0.02)		0.54*** (0.13)
Gender: Non-binary/Other		1.60 (6.61)		1.23 (5.45)		-0.57*** (0.13)		0.91 (0.79)
Education		2.56 (0.92)**		3.31*** (0.76)		0.05** (0.02)		0.07 (0.11)
Income		0.58 (0.37)		0.67* (0.31)		0.01 (0.01)		-0.02 (0.04)
Political Orientation		-0.39 (0.02)***		-0.24*** (0.02)		-0.00 (0.00)		-0.01*** (0.00)

†*p*<0.10, * *p<0.05, ** p<0.01, *** p<0.001*

Additional National-Level Covariates

The following tables represent the results of the models reported in main text but with different sets of controls. Each outcome is represented in a different table: Belief in Climate Change in Table S22, Climate Mitigation Policy Support in Table S23, Social Media Post in Table S24, Tree Planting Task in Table S25. For each of the tables, Model 1 represents the model without any covariates. Model 2 represents the model with the individual- and all the national-level covariates, and Model 3 includes also the national estimates of cultural individualism-collectivism. Compared to the pre-registered models, here we control for some additional national-level covariates: the Environmental Performance Index (Environmental Performance Index, 2024), the Vulnerability Index of Notre Dame Global Adaptation Initiative Country Index (Notre Dame Global Adaptation Initiative, 2023), the Gini Index (World Bank Data, 2025), and the Human Development Index (United Nations Human Development Report, 2015). These variables could plausibly interact with cultural tightness, so we include them to account for other omitted variables that might otherwise obscure or drive the observed effects.

Belief in Climate Change

We largely replicate the results found in the pre-registered model reported in Table 2. Specifically, we found that the interaction between the Pluralistic Ignorance condition and cultural tightness reached statistical significance. Similarly, the interaction between the Work-Together intervention and cultural tightness reached statistical significance as in the original model reported in Table 2, but for the model where we added the climate vulnerability as a covariate (p = .051).

Table S22.

Predicting Belief in Climate Change With Multiple Measures of National-Level Covariates.

Predictors	Model EPI Estimates (95%C.I.)	Model CV Estimates (95%C.I.)	Model GINI Estimates (95%C.I.)	Model HDI Estimates (95%C.I.)
Work-together Norm	-0.17 (-1.31 – 0.98)	-0.17 (-1.32 – 0.97)	-0.17 (-1.32 – 0.97)	-0.16 (-1.31 – 0.98)
Pluralistic Ignorance	0.91 (-0.23 – 2.05)	0.90 (-0.23 – 2.04)	0.91 (-0.23 – 2.05)	0.91 (-0.23 – 2.05)
Age	-0.09 *** (-0.12 – -0.06)	-0.09 *** (-0.12 – -0.06)	-0.09 *** (-0.12 – -0.06)	-0.09 *** (-0.12 – -0.06)
Gender: Female	4.16 *** (3.33 – 4.98)	4.17 *** (3.35 – 5.00)	4.17 *** (3.34 – 4.99)	4.16 *** (3.34 – 4.99)
Gender: Non-binary/other	4.28 (-0.89 – 9.46)	4.26 (-0.91 – 9.44)	4.30 (-0.88 – 9.47)	4.30 (-0.88 – 9.48)
Education	0.83 * (0.18 – 1.48)	0.81 * (0.16 – 1.46)	0.86 ** (0.21 – 1.51)	0.81 * (0.16 – 1.46)
Income	0.41 *** (0.17 – 0.66)	0.44 *** (0.19 – 0.68)	0.42 *** (0.17 – 0.66)	0.43 *** (0.19 – 0.67)
Political orientation	-0.22 *** (-0.24 – -0.21)	-0.22 *** (-0.24 – -0.21)	-0.22 *** (-0.24 – -0.21)	-0.22 *** (-0.24 – -0.21)
Emissions per capita	0.13 (-0.59 – 0.85)	0.19 (-0.48 – 0.86)	0.08 (-0.63 – 0.79)	0.17 (-0.47 – 0.82)
GDP per capita	0.00 (-0.94 – 0.95)	0.14 (-0.75 – 1.03)	-0.07 (-1.01 – 0.86)	-0.26 (-1.09 – 0.57)
Additional Covariate	-0.24 ** (-0.40 – -0.09)	68.76 *** (33.85 – 103.67)	0.44 ** (0.15 – 0.72)	-38.06 *** (-55.79 – -20.33)

Dynamic Norm * Tightness	-0.71<br>(-5.40 – 3.98)	-0.70<br>(-5.39 – 3.99)	-0.67<br>(-5.36 – 4.02)	-0.68<br>(-5.37 – 4.01)
Work-together Norm * Tightness	4.74 *<br>(0.01 – 9.47)	4.72<br>(-0.01 – 9.45)	4.76 *<br>(0.03 – 9.50)	4.75 *<br>(0.02 – 9.49)
Pluralistic Ignorance * Tightness	-5.88 *<br>(-10.59 – -1.16)	-5.88 *<br>(-10.59 – -1.16)	-5.84 *<br>(-10.55 – -1.12)	-5.82 *<br>(-10.54 – -1.11)
Random Effects				
σ2	521.16	521.18	521.17	521.18
τ00	25.11 Country	21.68 Country	25.13 Country	20.39 Country
ICC	0.05	0.04	0.05	0.04
N	39 Country	39 Country	39 Country	39 Country
Observations	12474	12474	12474	12474
Marginal R2 / Conditional R2	0.080 / 0.122	0.084 / 0.121	0.078 / 0.120	0.087 / 0.121

* p<0.05 ** p<0.01 *** p<0.001

Climate Mitigation Policy Support

In all four models, similarly to the pre-registered models, only the interaction between Pluralistic Ignorance intervention and cultural tightness reached statistical significance.

Table S23.

Predicting Climate Mitigation Policy Support With Multiple Measures of National-Level Covariates.

Predictors	Model EPI Estimates (95%C.I.)	Model CV Estimates (95%C.I.)	Model GINI Estimates (95%C.I.)	Model HDI Estimates (95%C.I.)
Work-together Norm	0.31 (-0.63 – 1.25)	0.30 (-0.64 – 1.24)	0.30 (-0.63 – 1.24)	0.31 (-0.63 – 1.25)
Pluralistic Ignorance	0.76 (-0.17 – 1.69)	0.76 (-0.17 – 1.69)	0.76 (-0.17 – 1.70)	0.76 (-0.17 – 1.70)
Age	0.00 (-0.02 – 0.02)	-0.00 (-0.02 – 0.02)	-0.00 (-0.03 – 0.02)	0.00 (-0.02 – 0.02)
Gender: Female	1.51 *** (0.83 – 2.19)	1.53 *** (0.85 – 2.20)	1.52 *** (0.84 – 2.20)	1.52 *** (0.84 – 2.20)
Gender: Non-binary/other	3.56 (-0.71 – 7.82)	3.55 (-0.72 – 7.82)	3.57 (-0.70 – 7.83)	3.59 (-0.68 – 7.85)
Education	1.40 *** (0.87 – 1.94)	1.40 *** (0.86 – 1.93)	1.43 *** (0.89 – 1.96)	1.39 *** (0.86 – 1.93)
Income	0.39 *** (0.19 – 0.59)	0.41 *** (0.21 – 0.61)	0.40 *** (0.20 – 0.60)	0.41 *** (0.21 – 0.61)
Political orientation	-0.14 *** (-0.16 – -0.13)	-0.14 *** (-0.16 – -0.13)	-0.14 *** (-0.15 – -0.13)	-0.14 *** (-0.16 – -0.13)
Emissions per capita	0.40 (-0.15 – 0.96)	0.31 (-0.29 – 0.92)	0.24 (-0.39 – 0.86)	0.34 (-0.21 – 0.89)
GDP per capita	-0.37 (-1.09 – 0.36)	-0.41 (-1.22 – 0.39)	-0.57 (-1.39 – 0.25)	-0.73 * (-1.44 – -0.03)
Additional Covariate	-0.30 *** (-0.42 – -0.18)	58.72 *** (27.27 – 90.16)	0.40 ** (0.15 – 0.65)	-36.25 *** (-51.29 – -21.22)
Dynamic Norm * Tightness	-1.15 (-4.99 – 2.69)	-1.13 (-4.98 – 2.71)	-1.11 (-4.95 – 2.73)	-1.12 (-4.96 – 2.73)
Work-together Norm * Tightness	3.18 (-0.69 – 7.06)	3.17 (-0.71 – 7.04)	3.20 (-0.67 – 7.08)	3.19 (-0.68 – 7.07)

Pluralistic Ignorance * Tightness	-4.14 * (-8.00 – -0.27)	-4.13 * (-7.99 – -0.27)	-4.10 * (-7.96 – -0.23)	-4.09 * (-7.95 – -0.23)
Random Effects				
σ2	349.25	349.26	349.26	349.27
τ00	14.67 Country	17.96 Country	19.55 Country	14.78 Country
ICC	0.04	0.05	0.05	0.04
N	39 Country	39 Country	39 Country	39 Country
Observations	12436	12436	12436	12436
Marginal R2 / Conditional R2	0.064 / 0.101	0.059 / 0.105	0.059 / 0.109	0.065 / 0.103

* p<0.05 ** p<0.01 *** p<0.001

Social Media Post Sharing Intention

In all three models, none of the three interactions reached statistical significance, as previously shown in the pre-registered analyses reported in Table 2.

Table S24.

Predicting Social Media Post Sharing Intention With Multiple Measures of National-Level Covariates.

Predictors	Model EPI Estimates (95%C.I.)	Model CV Estimates (95%C.I.)	Model GINI Estimates (95%C.I.)	Model HDI Estimates (95%C.I.)
Work-together Norm	0.05 *** (0.03 – 0.08)	0.05 *** (0.03 – 0.08)	0.05 *** (0.03 – 0.08)	0.05 *** (0.03 – 0.08)
Pluralistic Ignorance	0.02 (-0.01 – 0.05)	0.02 (-0.01 – 0.05)	0.02 (-0.01 – 0.05)	0.02 (-0.01 – 0.05)
Age	-0.00 *** (-0.00 – -0.00)	-0.00 *** (-0.00 – -0.00)	-0.00 *** (-0.00 – -0.00)	-0.00 *** (-0.00 – -0.00)
Gender: Female	-0.05 *** (-0.06 – -0.03)	-0.05 *** (-0.06 – -0.03)	-0.05 *** (-0.06 – -0.03)	-0.05 *** (-0.06 – -0.03)
Gender: Non-binary/other	-0.10 (-0.22 – 0.01)	-0.10 (-0.22 – 0.01)	-0.10 (-0.22 – 0.01)	-0.10 (-0.22 – 0.01)
Education	0.04 *** (0.02 – 0.05)	0.04 *** (0.02 – 0.05)	0.04 *** (0.03 – 0.06)	0.04 *** (0.02 – 0.05)
Income	0.00 (-0.00 – 0.01)	0.00 (-0.00 – 0.01)	0.00 (-0.00 – 0.01)	0.00 (-0.00 – 0.01)
Political orientation	0.00 *** (0.00 – 0.00)	0.00 *** (0.00 – 0.00)	0.00 *** (0.00 – 0.00)	0.00 *** (0.00 – 0.00)
Emissions per capita	0.00 (-0.02 – 0.03)	0.01 (-0.02 – 0.03)	0.00 (-0.02 – 0.03)	0.00 (-0.02 – 0.03)
GDP per capita	-0.03 (-0.06 – 0.00)	-0.03 (-0.06 – 0.00)	-0.03 * (-0.07 – -0.00)	-0.04 * (-0.07 – -0.01)
Additional Covariate	-0.01 ** (-0.01 – -0.00)	2.02 ** (0.82 – 3.23)	0.01 ** (0.00 – 0.02)	-0.86 * (-1.52 – -0.20)
Dynamic Norm * Tightness	0.02 (-0.09 – 0.12)	0.02 (-0.09 – 0.12)	0.02 (-0.09 – 0.12)	0.02 (-0.09 – 0.12)
Work-together Norm * Tightness	0.01 (-0.09 – 0.11)	0.01 (-0.09 – 0.11)	0.01 (-0.09 – 0.11)	0.01 (-0.09 – 0.11)
Pluralistic Ignorance * Tightness	-0.04 (-0.14 – 0.07)	-0.03 (-0.14 – 0.07)	-0.03 (-0.14 – 0.07)	-0.03 (-0.14 – 0.07)

Random Effects				
σ2	0.20	0.20	0.20	0.20
τ00	0.03 Country	0.03 Country	0.03 Country	0.03 Country
ICC	0.13	0.12	0.13	0.13
N	39 Country	39 Country	39 Country	39 Country
Observations	9537	9537	9537	9537
Marginal R2 / Conditional R2	0.084 / 0.202	0.092 / 0.201	0.088 / 0.206	0.084 / 0.205

* $p<0.05$ ** $p<0.01$ *** $p<0.001$

Tree Planting Task

In all four models, only the interaction between Pluralistic Ignorance intervention and cultural tightness reached statistical significance. This differs from the primary pre-registered model reported in Table 2, in which the interaction was not significant.

Table S25.

Predicting the Tree Planting Task With Multiple Measures of National-Level Covariates.

Predictors	Model EPI Estimates (95%C.I.)	Model CV Estimates (95%C.I.)	Model GINI Estimates (95%C.I.)	Model HDI Estimates (95%C.I.)
Work-together Norm	-0.37 *** (-0.53 – -0.21)	-0.37 *** (-0.53 – -0.21)	-0.37 *** (-0.53 – -0.21)	-0.37 *** (-0.53 – -0.21)
Pluralistic Ignorance	-0.13 (-0.29 – 0.03)	-0.13 (-0.29 – 0.03)	-0.13 (-0.29 – 0.03)	-0.13 (-0.29 – 0.03)
Age	0.04 *** (0.03 – 0.04)	0.04 *** (0.03 – 0.04)	0.04 *** (0.03 – 0.04)	0.04 *** (0.03 – 0.04)
Gender: Female	0.46 *** (0.35 – 0.58)	0.46 *** (0.35 – 0.58)	0.46 *** (0.35 – 0.58)	0.46 *** (0.35 – 0.58)
Gender: Non-binary/other	0.41 (-0.33 – 1.14)	0.41 (-0.33 – 1.14)	0.41 (-0.32 – 1.14)	0.41 (-0.32 – 1.14)
Education	0.05 (-0.04 – 0.14)	0.05 (-0.04 – 0.15)	0.05 (-0.04 – 0.15)	0.05 (-0.04 – 0.15)
Income	0.02 (-0.01 – 0.05)	0.02 (-0.01 – 0.06)	0.02 (-0.01 – 0.06)	0.02 (-0.01 – 0.06)
Political orientation	-0.01 *** (-0.01 – -0.01)	-0.01 *** (-0.01 – -0.01)	-0.01 *** (-0.01 – -0.01)	-0.01 *** (-0.01 – -0.01)
Emissions per capita	-0.11 * (-0.20 – -0.02)	-0.12 * (-0.21 – -0.03)	-0.12 ** (-0.21 – -0.03)	-0.12 * (-0.21 – -0.03)
GDP per capita	0.02 (-0.09 – 0.14)	0.01 (-0.11 – 0.13)	0.00 (-0.11 – 0.12)	0.00 (-0.11 – 0.12)
Additional Covariate	-0.01 (-0.03 – 0.01)	1.34 (-3.40 – 6.09)	0.01 (-0.03 – 0.04)	-0.77 (-3.24 – 1.70)
Dynamic Norm * Tightness	-0.06 (-0.72 – 0.60)	-0.06 (-0.72 – 0.60)	-0.06 (-0.72 – 0.60)	-0.06 (-0.72 – 0.60)
Work-together Norm * Tightness	-0.05 (-0.72 – 0.61)	-0.05 (-0.72 – 0.61)	-0.05 (-0.72 – 0.61)	-0.05 (-0.72 – 0.61)

Pluralistic Ignorance * Tightness	-0.73 * (-1.40 – -0.07)	-0.73 * (-1.40 – -0.07)	-0.73 * (-1.40 – -0.07)	-0.73 * (-1.40 – -0.07)
Random Effects				
σ2	0.20	0.20	0.20	0.20
τ00	0.03 $_{\text{Country}}$	0.03 $_{\text{Country}}$	0.03 $_{\text{Country}}$	0.03 $_{\text{Country}}$
ICC	0.13	0.12	0.13	0.13
N	39 $_{\text{Country}}$	39 $_{\text{Country}}$	39 $_{\text{Country}}$	39 $_{\text{Country}}$
Observations	9537	9537	9537	9537
Marginal R2 / Conditional R2	0.084 / 0.202	0.092 / 0.201	0.088 / 0.206	0.084 / 0.205

* p<0.05 ** p<0.01 *** p<0.001

Representativeness Checks

First, we checked the correlation between cultural tightness and representativeness. We ran a Spearman correlation between nation-level cultural tightness and representativeness, an ordinal variable with three values: 0 = countries with samples that were not representative in any dimension (e.g., Algeria or Czech Republic), 1 = countries with samples that were either representative only in one dimension (i.e., Australia) or that consisted of different samples with various levels of representativeness (e.g., Canada, that had a non-representative subsample of 858 participants and a subsample of 303 participants that were representative of the Canadian population no two dimensions), 2 = countries with samples that were representative in at least two dimensions (e.g., Belgium was representative on gender and age, and Slovakia was representative on age, gender, region and municipality size). We found that cultural tightness and representativeness are not significantly correlated ($\rho = -0.21$, $p = 0.18$).

Then we re-ran the analysis of the main model reported in Table 2 in the manuscript but dividing the sample in countries with low-representativeness (i.e., those countries with 0 or 1 value in the representativeness score) and high-representativeness (i.e., those countries with value 2). The following tables represent the results of these models, each table reports the results of one outcome: Belief in Climate Change in Table S26, Climate Mitigation Policy Support in Table S27, Social Media Post in Table S28, Tree Planting Task in Table S29. For each of the tables, Model 1 represents the model in countries with low-representativeness, and Model 2 the model in countries with high-representativeness.

Belief in Climate Change

Only in the model with low-representativeness (Model 1, Table S27), the interaction between Work-Together Norm condition and cultural tightness reached statistical significance, but not the interaction with Pluralistic Ignorance condition as found in the main analyses reported in Table 2.

Table S26.

Predicting Belief in Climate Change in Countries with Low- vs. High-Representativeness.

Predictors	Model 1 (Low-represent.) Estimates	Model 2 (High-represent.) Estimates
Work-together Norm	-1.20<br>(-2.70 – 0.30)	0.66<br>(-0.99 – 2.31)
Pluralistic Ignorance	1.61 *<br>(0.11 – 3.12)	0.37<br>(-1.26 – 2.01)
Age	-0.05 *<br>(-0.09 – -0.01)	-0.12 ***<br>(-0.16 – -0.08)
Gender: Female	4.22 ***<br>(3.12 – 5.32)	4.14 ***<br>(2.96 – 5.32)
Gender: Non-binary/other	0.85<br>(-5.20 – 6.91)	10.05 *<br>(1.41 – 18.68)
Education	0.47<br>(-0.42 – 1.36)	0.99 *<br>(0.07 – 1.90)
Income	-0.13<br>(-0.45 – 0.19)	0.88 ***<br>(0.53 – 1.24)
Political orientation	-0.18 ***<br>(-0.21 – -0.16)	-0.24 ***<br>(-0.27 – -0.22)
Emissions per capita	-0.20<br>(-0.96 – 0.56)	-0.26<br>(-1.80 – 1.27)
GDP per capita	-0.75<br>(-1.82 – 0.32)	-0.33<br>(-2.13 – 1.47)
Dynamic Norm * Tightness	-3.28<br>(-9.18 – 2.62)	2.57<br>(-4.14 – 9.29)
Work-together Norm * Tightness	6.43 *<br>(0.40 – 12.46)	3.43<br>(-3.26 – 10.12)
Pluralistic Ignorance * Tightness	-4.40<br>(-10.39 – 1.59)	-6.34<br>(-13.05 – 0.36)
**Random Effects**		
σ^2	414.56	603.25
τ_{00}	16.15 Country	58.31 Country
ICC	0.04	0.09
N	23 Country	18 Country
Observations	5786	6974
Marginal R^2 / Conditional R^2	0.067 / 0.102	0.069 / 0.151

* *p<0.05* ** *p<0.01* *** *p<0.001*

Climate Mitigation Policy Support

None of the interactions reached statistical significance in the Models divided by representativeness.

Table S27.
Predicting Climate Mitigation Policy Support in Countries with Low- vs. High-Representativeness.

Predictors	**Model 1 (Low-represent.)** *Estimates*	**Model 2 (High-represent.)** *Estimates*
Work-together Norm	-1.04 (-2.28 – 0.20)	1.23 (-0.12 – 2.58)
Pluralistic Ignorance	0.41 (-0.83 – 1.65)	0.93 (-0.41 – 2.26)
Age	0.04 ** (0.01 – 0.08)	-0.03 (-0.06 – 0.00)
Gender: Female	2.99 *** (2.08 – 3.90)	0.42 (-0.55 – 1.38)
Gender: Non-binary/other	3.01 (-2.04 – 8.06)	5.05 (-2.01 – 12.11)
Education	0.85 * (0.11 – 1.58)	1.71 *** (0.96 – 2.46)
Income	0.13 (-0.13 – 0.40)	0.59 *** (0.30 – 0.88)
Political orientation	-0.12 *** (-0.14 – -0.10)	-0.15 *** (-0.17 – -0.13)
Emissions per capita	0.29 (-0.53 – 1.11)	-0.41 (-1.54 – 0.71)
GDP per capita	-1.18 * (-2.33 – -0.03)	-0.43 (-1.75 – 0.90)
Dynamic Norm * Tightness	-2.08 (-6.95 – 2.79)	-1.54 (-7.04 – 3.96)
Work-together Norm * Tightness	2.25 (-2.74 – 7.23)	2.28 (-3.19 – 7.75)
Pluralistic Ignorance * Tightness	-4.19 (-9.14 – 0.76)	-5.17 (-10.65 – 0.32)
**Random Effects**		
σ^2	282.35	403.29
τ_{00}	19.50 Country	31.21 Country
ICC	0.06	0.07
N	23 Country	18 Country
Observations	5773	6949
Marginal R^2 / Conditional R^2	0.064 / 0.124	0.042 / 0.111

* *p<0.05* ** *p<0.01* *** *p<0.001*

Social Media Post Sharing Intention

In both models, none of the three interactions reached statistical significance, as previously shown in the pre-registered analyses (see Table 2).

Table S28.
Predicting Social Media Post Sharing Intention in Countries with Low- vs. High-Representativeness.

	**Model 1 (Low-represent.)**	**Model 2 (High-represent.)**
Predictors	*Estimates*	*Estimates*
Work-together Norm	0.06 ** (0.02 – 0.09)	0.05 ** (0.01 – 0.08)
Pluralistic Ignorance	0.01 (-0.02 – 0.05)	0.02 (-0.01 – 0.06)
Age	0.00 *** (0.00 – 0.00)	-0.00 *** (-0.01 – -0.00)
Gender: Female	-0.02 (-0.04 – 0.01)	-0.06 *** (-0.09 – -0.04)
Gender: Non-binary/other	0.07 (-0.09 – 0.23)	-0.32 *** (-0.50 – -0.15)
Education	0.01 (-0.01 – 0.03)	0.06 *** (0.04 – 0.08)
Income	0.00 (-0.00 – 0.01)	0.00 (-0.00 – 0.01)
Political orientation	0.00 *** (0.00 – 0.00)	0.00 (-0.00 – 0.00)
Emissions per capita	0.01 (-0.02 – 0.04)	-0.01 (-0.05 – 0.03)
GDP per capita	-0.02 (-0.06 – 0.02)	-0.05 * (-0.10 – -0.01)
Dynamic Norm * Tightness	0.04 (-0.11 – 0.18)	-0.03 (-0.16 – 0.10)
Work-together Norm * Tightness	-0.00 (-0.15 – 0.15)	0.02 (-0.11 – 0.15)
Pluralistic Ignorance * Tightness	0.06 (-0.08 – 0.21)	-0.13 (-0.27 – 0.01)
**Random Effects**		
σ^2	0.20	0.20
τ_{00}	0.03 Country	0.04 Country
ICC	0.11	0.16
N	23 Country	18 Country
Observations	4406	5388
Marginal R^2 / Conditional R^2	0.106 / 0.205	0.086 / 0.230

* *p<0.05* ** *p<0.01* *** *p<0.001*

Tree Planting Task

In both models, none of the three interactions reached statistical significance, as previously shown in the pre-registered analyses (see Table 2).

Table S29.

Predicting the Tree Planting Task in Countries with Low- vs. High-Representativeness.

	**Model 1 (Low-represent.)**	**Model 2 (High-represent.)**
Predictors	*Estimates*	*Estimates*
Work-together Norm	-0.50 ***	-0.27 *
	(-0.74 – -0.27)	(-0.49 – -0.05)
Pluralistic Ignorance	-0.17	-0.07
	(-0.40 – 0.07)	(-0.28 – 0.15)
Age	0.03 ***	0.04 ***
	(0.03 – 0.04)	(0.04 – 0.05)
Gender: Female	0.51 ***	0.44 ***
	(0.34 – 0.68)	(0.28 – 0.60)
Gender: Non-binary/other	0.39	0.47
	(-0.56 – 1.34)	(-0.67 – 1.61)
Education	0.11	0.02
	(-0.03 – 0.25)	(-0.10 – 0.14)
Income	0.01	0.03
	(-0.04 – 0.06)	(-0.01 – 0.08)
Political orientation	-0.01 ***	-0.01 ***
	(-0.01 – -0.01)	(-0.01 – -0.01)
Emissions per capita	-0.08	-0.17 **
	(-0.20 – 0.04)	(-0.30 – -0.04)
GDP per capita	0.08	-0.02
	(-0.09 – 0.25)	(-0.17 – 0.13)
Dynamic Norm * Tightness	0.42	-0.43
	(-0.50 – 1.35)	(-1.32 – 0.45)
Work-together Norm * Tightness	-0.11	-0.11
	(-1.06 – 0.83)	(-1.00 – 0.77)
Pluralistic Ignorance * Tightness	-0.68	-0.49
	(-1.62 – 0.26)	(-1.38 – 0.39)
**Random Effects**		
σ^2	10.19	10.58
τ_{00}	0.41 $_{\text{Country}}$	0.38 $_{\text{Country}}$
ICC	0.04	0.03
N	23 $_{\text{Country}}$	18 $_{\text{Country}}$
Observations	5788	6981
Marginal R^2 / Conditional R^2	0.047 / 0.084	0.055 / 0.088

* *p<0.05* ** *p<0.01* *** *p<0.001*

Cumulative CO_2 Emissions Median Split

Given that climate change responsibility is related to the CO_2 emissions, in the following tables we report the results of the main model reported in Table 2 but dividing the sample in countries based on the median of the cumulative CO_2 emissions, so into countries with historically lower CO_2 emissions (i.e., below the median) and historically higher CO_2 emissions (i.e., above the median). The following tables represent the results of these models, each table reports the results of one outcome: Belief in Climate Change in Table S30, Climate Mitigation Policy Support in Table S31, Social Media Post in Table S32, Tree Planting Task in Table S33. For each of the tables, Model 1 represents the model in countries with cumulatively low emissions of CO_2 –which limitedly impacted climate change–and Model 2 the model in countries with cumulatively high emissions of CO_2–which are the most responsible for climate change. The results show that none of the interactions is significant when dividing the sample based on countries' emissions, the only difference is for the interaction of the Pluralistic Ignorance condition and cultural tightness on the belief in climate change and the tree-planting task. Specifically, this interaction reached statistical significance only in the high emitting countries for belief (see Table S30), while only in little CO_2 emitting countries for the tree-planting task (see Table S33).

Belief in Climate Change

None of the interactions reached statistical significance in Model 1 considering low emitting countries, while in Model 2 –considering those countries with higher cumulative CO_2 emissions– the interaction between the Pluralistic Ignorance condition and cultural tightness reached statistical significance.

Table S30.

Predicting Belief in Climate Change in Countries with Low- vs. High-Emissions.

Predictors	**Model 1 (low-emis.)** *Estimates (95% C.I.)*	**Model 2 (high-emis.)** *Estimates (95% C.I.)*
Intercept	73.97*** (39.25 – 108.70)	56.71 ** (21.21 – 92.22)
Cultural Tightness	-0.38 (-12.60 – 11.83)	4.88 (-10.80 – 20.56)
Dynamic Norm	-0.81 (-2.85 – 1.23)	1.31 (-0.17 – 2.80)
Work-together Norm	0.12 (-1.92 – 2.17)	-0.45 (-1.95 – 1.05)
Pluralistic Ignorance	-0.76 (-2.79 – 1.26)	1.35 (-0.15 – 2.84)
Age	0.03 (-0.02 – 0.09)	-0.13 *** (-0.16 – -0.09)
Gender: Female	4.78 *** (3.40 – 6.16)	3.83 *** (2.80 – 4.85)
Gender: Non-binary/other	9.35 (-0.37 – 19.06)	1.41 (-4.70 – 7.51)
Education	0.51 (-0.61 – 1.62)	0.93 * (0.14 – 1.73)
Income	0.70 *** (0.32 – 1.08)	0.23 (-0.09 – 0.55)
Political orientation	-0.09 *** (-0.12 – -0.06)	-0.28 *** (-0.30 – -0.26)
Emissions per capita	0.41 (-1.04 – 1.86)	0.34 (-0.53 – 1.20)
Environmental Performance	-0.01 (-0.34 – 0.33)	0.03 (-0.33 – 0.39)
Vulnerability	14.80 (-76.23 – 105.84)	60.83 (-36.05 – 157.71)
GDP per capita	-0.55 (-2.42 – 1.32)	0.61 (-0.80 – 2.01)
Gini index	0.20 (-0.38 – 0.78)	0.39 * (0.03 – 0.74)
Human Development Index	-36.94 (-93.12 – 19.24)	-7.59 (-56.82 – 41.65)
Dynamic Norm * Tightness	5.04 (-1.18 – 11.26)	-5.58 (-13.54 – 2.37)
Work-together Norm * Tightness	5.54 (-0.76 – 11.84)	1.38 (-6.70 – 9.47)

Pluralistic Ignorance * Tightness	-1.06<br>(-7.33 – 5.22)	-9.21 *<br>(-17.24 – -1.18)
**Random Effects**		
σ^2	479.37	534.28
τ_{00}	38.74 $_{Country}$	18.46 $_{Country}$
ICC	0.07	0.03
N	20 $_{Country}$	19 $_{Country}$
Observations	4156	8318
Marginal R^2 / Conditional R^2	0.070 / 0.139	0.112 / 0.142

* *p<0.05* ** *p<0.01* *** *p<0.001*

Climate Mitigation Policy Support

In both sub-samples, none of the three interactions reached statistical significance.

Table S31.

Predicting Climate Mitigation Policies Support in Countries with Low- vs. High-Emissions.

Predictors	**Model 1 (low-emis.)**<br>*Estimates*<br>*(95% C.I.)*	**Model 2 (high-emis.)**<br>*Estimates*<br>*(95% C.I.)*
Intercept	72.67 ***<br>(52.50 – 92.84)	61.66 **<br>(22.57 – 100.74)
Cultural Tightness	2.89<br>(-4.50 – 10.27)	4.33<br>(-12.55 – 21.21)
Dynamic Norm	1.11<br>(-0.54 – 2.76)	1.21<br>(-0.02 – 2.44)
Work-together Norm	0.62<br>(-1.04 – 2.27)	-0.06<br>(-1.30 – 1.18)
Pluralistic Ignorance	0.82<br>(-0.82 – 2.46)	0.63<br>(-0.60 – 1.87)
Age	0.09 ***<br>(0.05 – 0.14)	-0.03<br>(-0.05 – 0.00)
Gender: Female	1.98 ***<br>(0.87 – 3.09)	1.27 **<br>(0.42 – 2.12)
Gender: Non-binary/other	3.80<br>(-4.04 – 11.64)	2.50<br>(-2.58 – 7.59)
Education	0.99 *<br>(0.10 – 1.89)	1.51 ***<br>(0.85 – 2.17)
Income	0.35 *<br>(0.04 – 0.65)	0.43 **<br>(0.16 – 0.69)
Political orientation	-0.04 ***<br>(-0.07 – -0.02)	-0.18 ***<br>(-0.20 – -0.16)
Emissions per capita	0.58<br>(-0.26 – 1.41)	0.49<br>(-0.45 – 1.44)
Environmental Performance	-0.22 *<br>(-0.41 – -0.03)	-0.00<br>(-0.40 – 0.40)
Vulnerability	-6.00<br>(-58.83 – 46.83)	26.78<br>(-79.91 – 133.47)
GDP per capita	-0.26<br>(-1.34 – 0.82)	-0.48<br>(-2.03 – 1.07)
Gini index	0.31<br>(-0.02 – 0.65)	0.22<br>(-0.17 – 0.61)
Human Development Index	-12.25<br>(-44.79 – 20.28)	-21.99<br>(-75.97 – 32.00)
Dynamic Norm * Tightness	0.88<br>(-4.14 – 5.90)	-3.91<br>(-10.48 – 2.66)
Work-together Norm * Tightness	4.37<br>(-0.72 – 9.45)	-0.25<br>(-6.93 – 6.43)
Pluralistic Ignorance * Tightness	-2.93<br>(-8.00 – 2.13)	-5.97<br>(-12.61 – 0.66)
**Random Effects**		
σ^2	312.36	363.83
τ_{00}	11.98 $_{Country}$	23.44 $_{Country}$
ICC	0.04	0.06

N	20 Country	19 Country
Observations	4149	8287
Marginal R^2 / Conditional R^2	0.072 / 0.106	0.077 / 0.133

* *p<0.05* ** *p<0.01* *** *p<0.001*

Social Media Post Sharing Intention

In both sub-samples, none of the three interactions reached statistical significance.

Table S32.

Predicting Social Media Post Sharing Intention in Countries with Low- vs. High-Emissions.

Predictors	**Model 1 (low-emis.)** *Estimates (95% C.I.)*	**Model 2 (high-emis.)** *Estimates (95% C.I.)*
Intercept	0.00 (-0.74 – 0.74)	-0.08 (-1.60 – 1.44)
Cultural Tightness	-0.01 (-0.27 – 0.25)	-0.01 (-0.66 – 0.63)
Dynamic Norm	0.08 *** (0.03 – 0.13)	0.07 *** (0.04 – 0.11)
Work-together Norm	0.04 (-0.01 – 0.08)	0.05 ** (0.01 – 0.08)
Pluralistic Ignorance	0.02 (-0.03 – 0.07)	0.03 (-0.01 – 0.06)
Age	0.00 (-0.00 – 0.00)	-0.00 *** (-0.00 – -0.00)
Gender: Female	-0.02 (-0.05 – 0.01)	-0.06 *** (-0.09 – -0.04)
Gender: Non-binary/other	0.06 (-0.17 – 0.29)	-0.17 * (-0.31 – -0.03)
Education	0.04 ** (0.01 – 0.06)	0.04 *** (0.02 – 0.06)
Income	-0.01 (-0.01 – 0.00)	0.01 ** (0.00 – 0.02)
Political orientation	0.00 *** (0.00 – 0.00)	0.00 (-0.00 – 0.00)
Emissions per capita	0.04 * (0.01 – 0.07)	-0.01 (-0.05 – 0.02)
Environmental Performance	-0.00 (-0.01 – 0.01)	-0.00 (-0.02 – 0.01)
Vulnerability	1.55 (-0.39 – 3.48)	1.67 (-2.48 – 5.81)
GDP per capita	-0.05 * (-0.09 – -0.01)	0.01 (-0.05 – 0.07)
Gini index	0.00 (-0.01 – 0.02)	0.01 (-0.00 – 0.03)
Human Development Index	-0.54 (-1.74 – 0.65)	0.16 (-1.93 – 2.26)
Dynamic Norm * Tightness	-0.02 (-0.15 – 0.12)	0.06 (-0.11 – 0.24)
Work-together Norm * Tightness	0.08 (-0.06 – 0.21)	-0.07 (-0.25 – 0.10)
Pluralistic Ignorance * Tightness	-0.09 (-0.23 – 0.05)	0.05 (-0.12 – 0.23)
**Random Effects**		
σ^2	0.19	0.21
τ_{00}	0.02 $_{Country}$	0.04 $_{Country}$
ICC	0.08	0.15

N	20 $_{Country}$	19 $_{Country}$
Observations	3261	6276
Marginal R^2 / Conditional R^2	0.167 / 0.237	0.082 / 0.217

* *p<0.05* ** *p<0.01* *** *p<0.001*

Tree Planting Task

None of the interactions reached statistical significance in Model 2 considering historically highly emitting countries, while in Model 1 –considering those countries with low CO_2 emissions– the interaction between the Pluralistic Ignorance condition and cultural tightness reached statistical significance.

Table S33.

Predicting the Tree Planting Task in Countries with Low- vs. High-Emissions.

Predictors	**Model 1 (low-emis.)** *Estimates (95% C.I.)*	**Model 2 (high-emis.)** *Estimates (95% C.I.)*
Intercept	3.37 * (0.07 – 6.68)	5.94 * (0.46 – 11.42)
Cultural Tightness	0.24 (-0.99 – 1.47)	-0.87 (-3.26 – 1.53)
Dynamic Norm	0.01 (-0.29 – 0.31)	-0.07 (-0.28 – 0.14)
Work-together Norm	-0.10 (-0.40 – 0.20)	-0.44 *** (-0.65 – -0.23)
Pluralistic Ignorance	0.12 (-0.18 – 0.41)	-0.22 * (-0.43 – -0.02)
Age	0.04 *** (0.03 – 0.05)	0.04 *** (0.03 – 0.04)
Gender: Female	0.42 *** (0.22 – 0.62)	0.49 *** (0.34 – 0.63)
Gender: Non-binary/other	0.80 (-0.63 – 2.23)	0.27 (-0.59 – 1.12)
Education	0.04 (-0.12 – 0.21)	0.06 (-0.05 – 0.17)
Income	0.04 (-0.01 – 0.10)	0.01 (-0.04 – 0.05)
Political orientation	-0.01 *** (-0.02 – -0.01)	-0.01 *** (-0.01 – -0.01)
Emissions per capita	-0.18 ** (-0.32 – -0.05)	-0.05 (-0.18 – 0.08)
Environmental Performance	-0.02 (-0.05 – 0.01)	-0.03 (-0.09 – 0.02)
Vulnerability	1.99 (-6.66 – 10.64)	-4.81 (-19.76 – 10.13)
GDP per capita	-0.07 (-0.25 – 0.10)	0.04 (-0.17 – 0.26)
Gini index	-0.03 (-0.08 – 0.03)	0.00 (-0.05 – 0.06)
Human Development Index	-0.20 (-5.52 – 5.12)	2.79 (-4.79 – 10.37)
Dynamic Norm * Tightness	-0.49 (-1.41 – 0.42)	0.51 (-0.60 – 1.62)
Work-together Norm * Tightness	-0.74 (-1.66 – 0.19)	0.43 (-0.70 – 1.56)

Pluralistic Ignorance * Tightness	-1.22 **<br>(-2.14 – -0.29)	-0.59<br>(-1.71 – 0.54)
**Random Effects**		
σ^2	10.35	10.43
τ_{00}	0.31 $_{Country}$	0.45 $_{Country}$
ICC	0.03	0.04
N	20 $_{Country}$	19 $_{Country}$
Observations	4160	8323
Marginal R^2 / Conditional R^2	0.063 / 0.090	0.049 / 0.089

* *p<0.05* ** *p<0.01* *** *p<0.001*

GDP per Capita Median Split

Given that the capacity to prevent and respond to the impacts of climate change is related to a country's GDP, in the following tables we report the results of the main model reported in Table 2 but dividing the sample in countries with low GDP per capita (i.e., below the median) and high GDP per capita (i.e., above the median). The following tables represent the results of these models, each table reports the results of one outcome: Belief in Climate Change in Table S34, Climate Mitigation Policy Support in Table S35, Social Media Post in Table S36, Tree Planting Task in Table S37. For each of the tables, Model 1 represents the model in countries with low GDP per capita, and Model 2 the model in countries with high GDP per capita. The results show that none of the interactions is significant when dividing the sample based on countries' GDP per capita but in two cases: the interaction between the Pluralistic Ignorance condition and cultural tightness reached statistical significance in those countries with low (but not with high) GDP per capita in the models on the belief in climate change (see Table S34), while the opposite was found in the models on the climate mitigation policy support where this interaction was significant in high GDP per capita countries but not in those countries with low GDP per capita (see Table S35).

Belief in Climate Change

None of the interactions reached statistical significance in Model 2 considering countries with higher GDP per capita, while in Model 1—considering those countries with lower GDP per capita—the interaction between the Pluralistic Ignorance condition and cultural tightness reached statistical significance.

Table S34.

Predicting Belief in Climate Change in Countries with Low vs. High GDP per Capita.

Predictors	**Model 1 (low GDP)** *Estimates (95% C.I.)*	**Model 2 (high GDP)** *Estimates (95% C.I.)*
Intercept	69.31 ***	106.86 ***
	(46.92 – 91.71)	(51.90 – 161.82)
Cultural Tightness	2.88	19.22
	(-6.74 – 12.49)	(-5.64 – 44.08)
Dynamic Norm	1.87 *	0.08
	(0.27 – 3.47)	(-1.54 – 1.71)
Work-together Norm	1.69 *	-2.48 **
	(0.08 – 3.30)	(-4.10 – -0.85)
Pluralistic Ignorance	2.54 **	-0.94
	(0.95 – 4.14)	(-2.56 – 0.68)
Age	-0.15 ***	0.00
	(-0.19 – -0.12)	(-0.04 – 0.05)
Gender: Female	4.20 ***	4.42 ***
	(3.05 – 5.35)	(3.24 – 5.60)
Gender: Non-binary/other	6.22	0.98
	(-0.67 – 13.11)	(-6.86 – 8.83)
Education	1.26 **	0.25
	(0.35 – 2.18)	(-0.66 – 1.17)
Income	0.37 *	0.61 ***
	(0.03 – 0.71)	(0.26 – 0.96)
Political orientation	-0.25 ***	-0.18 ***
	(-0.27 – -0.23)	(-0.21 – -0.15)
Emissions per capita	0.07	0.17
	(-1.00 – 1.14)	(-0.95 – 1.29)
Environmental Performance	-0.11	0.16
	(-0.36 – 0.14)	(-0.32 – 0.63)
Vulnerability	27.29	-65.92
	(-29.83 – 84.40)	(-207.30 – 75.45)
GDP per capita	1.42	-2.21
	(-3.91 – 6.75)	(-6.23 – 1.81)
Gini index	0.30 *	0.16
	(0.01 – 0.59)	(-0.66 – 0.98)
Human Development Index	-4.64	-59.91
	(-44.59 – 35.31)	(-124.82 – 5.01)
Dynamic Norm * Tightness	-1.15	-1.08
	(-7.02 – 4.71)	(-9.15 – 7.00)
Work-together Norm * Tightness	3.34	5.51
	(-2.63 – 9.31)	(-2.45 – 13.48)

Pluralistic Ignorance * Tightness	-7.51 *<br>(-13.44 – -1.57)	-4.11<br>(-12.09 – 3.88)
**Random Effects**		
σ^2	561.03	466.61
τ_{00}	12.54 $_{\text{Country}}$	33.65 $_{\text{Country}}$
ICC	0.02	0.07
N	20 $_{\text{Country}}$	19 $_{\text{Country}}$
Observations	6902	5572
Marginal R^2 / Conditional R^2	0.112 / 0.132	0.071 / 0.133

* *p<0.05* ** *p<0.01* *** *p<0.001*

Climate Mitigation Policy Support

None of the interactions reached statistical significance in Model 1 considering countries with lower GDP per capita, while in Model 2 –considering those countries with higher GDP per capita– the interaction between the Pluralistic Ignorance condition and cultural tightness reached statistical significance.

Table S35.

Predicting Climate Mitigation Policies Support in Countries with Low vs. High GDP per Capita.

Predictors	**Model 1 (low GDP)**<br>*Estimates*<br>*(95% C.I.)*	**Model 2 (high GDP)**<br>*Estimates*<br>*(95% C.I.)*
Intercept	69.54 ***<br>(52.81 – 86.27)	96.17 ***<br>(50.40 – 141.93)
Cultural Tightness	0.21<br>(-7.06 – 7.49)	15.94<br>(-4.74 – 36.61)
Dynamic Norm	1.80 **<br>(0.48 – 3.12)	0.78<br>(-0.53 – 2.10)
Work-together Norm	0.82<br>(-0.51 – 2.14)	-0.46<br>(-1.77 – 0.86)
Pluralistic Ignorance	1.69 *<br>(0.37 – 3.00)	-0.38<br>(-1.69 – 0.94)
Age	-0.05 **<br>(-0.08 – -0.02)	0.08 ***<br>(0.05 – 0.11)
Gender: Female	1.14 *<br>(0.19 – 2.09)	2.30 ***<br>(1.35 – 3.26)
Gender: Non-binary/other	4.94<br>(-0.74 – 10.63)	0.69<br>(-5.76 – 7.14)
Education	1.81 ***<br>(1.06 – 2.57)	0.80 *<br>(0.06 – 1.54)
Income	0.42 **<br>(0.14 – 0.71)	0.45 **<br>(0.17 – 0.74)
Political orientation	-0.16 ***<br>(-0.18 – -0.15)	-0.10 ***<br>(-0.12 – -0.08)
Emissions per capita	0.55<br>(-0.23 – 1.34)	0.22<br>(-0.71 – 1.16)
Environmental Performance	-0.21 *<br>(-0.40 – -0.03)	-0.04<br>(-0.43 – 0.36)
Vulnerability	5.17<br>(-37.52 – 47.86)	-66.31<br>(-184.02 – 51.41)
GDP per capita	0.25<br>(-3.72 – 4.22)	-1.14<br>(-4.49 – 2.21)
Gini index	0.18<br>(-0.03 – 0.40)	0.36<br>(-0.32 – 1.04)
Human Development Index	-11.59<br>(-41.56 – 18.37)	-44.80<br>(-98.87 – 9.27)
Dynamic Norm * Tightness	-0.62<br>(-5.47 – 4.23)	-3.04<br>(-9.59 – 3.50)
Work-together Norm * Tightness	4.15<br>(-0.78 – 9.08)	0.47<br>(-5.99 – 6.92)

Pluralistic Ignorance * Tightness	-3.20 (-8.10 – 1.70)	-7.07 * (-13.54 – -0.61)
**Random Effects**		
σ^2	382.07	305.40
τ_{00}	6.59 Country	23.43 Country
ICC	0.02	0.07
N	20 Country	19 Country
Observations	6884	5552
Marginal R^2 / Conditional R^2	0.083 / 0.098	0.062 / 0.129

* *p<0.05* ** *p<0.01* *** *p<0.001*

Social Media Post Sharing Intention

In both sub-samples, none of the three interactions reached statistical significance.

Table S36.

Predicting Social Media Post Sharing Intention in Countries with Low vs. High GDP per Capita.

Predictors	**Model 1 (low GDP)**<br>*Estimates*<br>*(95% C.I.)*	**Model 2 (high GDP)**<br>*Estimates*<br>*(95% C.I.)*
Intercept	0.18<br>(-0.75 – 1.10)	-0.29<br>(-2.16 – 1.58)
Cultural Tightness	0.11<br>(-0.27 – 0.49)	-0.05<br>(-0.88 – 0.79)
Dynamic Norm	0.07 ***<br>(0.04 – 0.11)	0.07 ***<br>(0.03 – 0.11)
Work-together Norm	0.05 **<br>(0.01 – 0.08)	0.05 **<br>(0.01 – 0.09)
Pluralistic Ignorance	0.04 *<br>(0.00 – 0.07)	-0.00<br>(-0.04 – 0.04)
Age	-0.00 ***<br>(-0.00 – -0.00)	0.00 *<br>(0.00 – 0.00)
Gender: Female	-0.05 ***<br>(-0.07 – -0.02)	-0.03 *<br>(-0.06 – -0.01)
Gender: Non-binary/other	-0.10<br>(-0.26 – 0.05)	-0.10<br>(-0.28 – 0.09)
Education	0.04 ***<br>(0.02 – 0.06)	0.03 **<br>(0.01 – 0.06)
Income	0.01 *<br>(0.00 – 0.02)	-0.00<br>(-0.01 – 0.01)
Political orientation	0.00 ***<br>(0.00 – 0.00)	0.00 *<br>(0.00 – 0.00)
Emissions per capita	0.00<br>(-0.05 – 0.05)	0.03<br>(-0.01 – 0.06)
Environmental Performance	-0.00<br>(-0.01 – 0.01)	-0.01<br>(-0.03 – 0.01)
Vulnerability	0.95<br>(-1.42 – 3.31)	2.24<br>(-2.56 – 7.04)
GDP per capita	-0.04<br>(-0.26 – 0.18)	0.02<br>(-0.12 – 0.15)
Gini index	0.01<br>(-0.00 – 0.02)	0.00<br>(-0.02 – 0.03)
Human Development Index	0.02<br>(-1.61 – 1.66)	0.28<br>(-1.93 – 2.50)
Dynamic Norm * Tightness	0.01<br>(-0.12 – 0.13)	0.03<br>(-0.15 – 0.21)
Work-together Norm * Tightness	0.00<br>(-0.12 – 0.13)	0.03<br>(-0.15 – 0.21)
Pluralistic Ignorance * Tightness	-0.03<br>(-0.16 – 0.09)	-0.04<br>(-0.22 – 0.14)
**Random Effects**		
σ^2	0.21	0.19
τ_{00}	0.03 Country	0.04 Country
ICC	0.11	0.17

N	20 Country	19 Country
Observations	5316	4221
Marginal R^2 / Conditional R^2	0.059 / 0.161	0.151 / 0.299

* *p<0.05* ** *p<0.01* *** *p<0.001*

Tree Planting Task

In both sub-samples, none of the three interactions reached statistical significance.

Table S37.

Predicting the Tree Planting Task in Countries with Low vs. High GDP per Capita.

Predictors	**Model 1 (low GDP)** *Estimates (95% C.I.)*	**Model 2 (high GDP)** *Estimates (95% C.I.)*
Intercept	4.68 * (0.24 – 9.12)	6.17 * (0.51 – 11.82)
Cultural Tightness	0.09 (-1.76 – 1.94)	0.36 (-2.25 – 2.97)
Dynamic Norm	0.00 (-0.22 – 0.22)	-0.17 (-0.41 – 0.07)
Work-together Norm	-0.37 *** (-0.59 – -0.15)	-0.35 ** (-0.59 – -0.11)
Pluralistic Ignorance	-0.11 (-0.33 – 0.10)	-0.14 (-0.38 – 0.10)
Age	0.04 *** (0.03 – 0.04)	0.04 *** (0.03 – 0.05)
Gender: Female	0.54 *** (0.38 – 0.69)	0.39 *** (0.21 – 0.56)
Gender: Non-binary/other	0.61 (-0.33 – 1.55)	0.03 (-1.13 – 1.20)
Education	0.11 (-0.01 – 0.23)	-0.01 (-0.14 – 0.13)
Income	0.02 (-0.02 – 0.07)	0.02 (-0.03 – 0.07)
Political orientation	-0.01 *** (-0.02 – -0.01)	-0.01 ** (-0.01 – -0.00)
Emissions per capita	-0.13 (-0.35 – 0.08)	-0.08 (-0.19 – 0.04)
Environmental Performance	-0.03 (-0.07 – 0.02)	-0.03 (-0.08 – 0.02)
Vulnerability	-1.64 (-12.95 – 9.66)	-4.27 (-18.82 – 10.28)
GDP per capita	-0.05 (-1.11 – 1.01)	-0.17 (-0.58 – 0.24)
Gini index	0.02 (-0.04 – 0.08)	-0.10 * (-0.18 – -0.01)
Human Development Index	1.97 (-5.88 – 9.82)	-1.65 (-8.29 – 4.99)
Dynamic Norm * Tightness	-0.15 (-0.95 – 0.65)	-0.02 (-1.22 – 1.18)
Work-together Norm * Tightness	-0.47 (-1.29 – 0.34)	0.79 (-0.39 – 1.98)
Pluralistic Ignorance * Tightness	-0.74 (-1.54 – 0.07)	-0.73 (-1.92 – 0.46)
**Random Effects**		
σ^2	10.44	10.34
τ_{00}	0.56 $_{Country}$	0.33 $_{Country}$
ICC	0.05	0.03

N	20 $_{Country}$	19 $_{Country}$
Observations	6908	5575
Marginal R^2 / Conditional R^2	0.067 / 0.115	0.051 / 0.081

* *p<0.05*   ** *p<0.01*   *** *p<0.001*

Cumulative CO_2 Emissions Interaction

Building on the results of the previous analyses (Tables S30-S33), we tested a 3-way interaction between cultural tightness, the Dynamic Norm intervention, and the cumulative CO_2 emissions of each country, while controlling for the individual-level covariates. This analysis allows us to test whether countries emissions moderate the effect of the Dynamic Norm condition given the fact that this intervention showed data on different countries, thus people might have been influenced based on the comparison groups in their condition and this might be relevant given the disproportionate responsibility in climate change of each country and the consequences they are facing. The following table represents the results of these models, i.e., the results for each outcome. The results show that none of the 3-way interactions reaches statistical significance.

Table 38.

Predicting Climate Change Attitudes and Behavior with the 3-way Interaction Between Cultural Tightness, Dynamic Norm Intervention, and Cumulative CO_2 Emissions, While Controlling for the Individual-Level Covariates.

Predictors	**Belief in Climate Change** *Estimates (95% C.I.)*	**Policy Support** *Estimates (95% C.I.)*	**Social Media Post** *Estimates (95% C.I.)*	**Tree Planting** *Estimates (95% C.I.)*
Intercept	77.43 *** (75.41 – 79.44)	69.29 *** (67.52 – 71.05)	0.51 *** (0.45 – 0.58)	4.32 *** (4.02 – 4.61)
Cultural Tightness	11.17 ** (3.75 – 18.59)	9.80 ** (3.22 – 16.37)	0.30 * (0.04 – 0.56)	0.46 (-0.63 – 1.55)
Dynamic Norm	0.71 (-0.59 – 2.00)	1.02 (-0.04 – 2.07)	0.07 *** (0.04 – 0.10)	-0.14 (-0.32 – 0.04)
Cumulative Emissions	-1.45 (-3.65 – 0.74)	-1.15 (-3.12 – 0.82)	0.01 (-0.07 – 0.09)	-0.04 (-0.36 – 0.29)
Age	-0.12 *** (-0.16 – -0.08)	-0.01 (-0.04 – 0.02)	-0.00 *** (-0.00 – -0.00)	0.04 *** (0.03 – 0.05)
Gender: Female	4.04 *** (2.85 – 5.23)	1.71 *** (0.74 – 2.68)	-0.05 *** (-0.08 – -0.02)	0.34 *** (0.18 – 0.51)
Gender: Non-binary/other	4.21 (-3.50 – 11.93)	6.06 (-0.21 – 12.33)	-0.10 (-0.28 – 0.07)	0.51 (-0.56 – 1.57)
Education	1.05 * (0.11 – 1.98)	1.76 *** (1.00 – 2.53)	0.04 *** (0.02 – 0.06)	0.09 (-0.04 – 0.21)
Income	0.42 * (0.07 – 0.77)	0.37 * (0.09 – 0.66)	0.00 (-0.01 – 0.01)	0.04 (-0.01 – 0.09)
Political orientation	-0.22 *** (-0.24 – -0.19)	-0.14 *** (-0.16 – -0.12)	0.00 *** (0.00 – 0.00)	-0.01 *** (-0.01 – -0.01)
Dynamic Norm * Tightness	-1.22 (-6.28 – 3.85)	-1.43 (-5.55 – 2.69)	0.03 (-0.08 – 0.14)	-0.03 (-0.74 – 0.67)
Tightness * Cumulative Emissions	10.49 (-6.29 – 27.27)	7.84 (-7.10 – 22.78)	0.08 (-0.52 – 0.67)	0.04 (-2.43 – 2.51)
Dynamic Norm * Cumulative Emissions	-0.11 (-1.14 – 0.93)	0.04 (-0.80 – 0.88)	0.01 (-0.02 – 0.03)	0.05 (-0.09 – 0.20)
Dynamic Norm * Tightness * Cumulative Emissions	-6.90 (-17.12 – 3.32)	-5.39 (-13.71 – 2.92)	0.11 (-0.10 – 0.32)	-0.19 (-1.61 – 1.22)
**Random Effects**				
σ^2	539.19	355.96	0.21	10.34
τ_{00}	26.64 Country	22.02 Country	0.04 Country	0.60 Country
ICC	0.05	0.06	0.16	0.05
N	39 Country	39 Country	39 Country	39 Country
Observations	6193	6172	4719	6200
Marginal R^2 / Conditional R^2	0.100 / 0.142	0.067 / 0.121	0.047 / 0.201	0.039 / 0.091

* *p<0.05* ** *p<0.01* *** *p<0.001*

Correlations Between National-Level Variables

Here we report the correlations between the national level variables from our primary and supplemental analyses: cultural tightness (our main moderator), CO_2 emissions per capita and GDP per capita (the two pre-registered national-level covariates), the Environmental Performance Index (EPI), the Climate Vulnerability Index of Notre Dame Global Adaptation Initiative Country Index (CV), the Gini index (GINI), the Human Development Index (HDI), historical cumulative CO_2 emissions used in our supplemental analyses reported in Tables S30-33 and S38, and individualism.

Table S39.

Correlations between National-Level Variables.

Variable	1	2	3	4	5	6	7	8
1. Cultural tightness								
2. CO_2 emissions per capita	-.27							
3. GDP per capita	-.17	.05						
4. EPI	-.54**	.40*	.33*					
5. CV (ND-GAIN)	.57**	-.40*	-.35*	-.79**				
6. GINI	.04	-.29	-.24	-.41**	.35*			
7. HDI	-.49**	.38*	.17	.78**	-.81**	-.35*		
8. Historical CO_2 emissions	-.07	-.14	-.07	-.02	-.13	.13	.11	
9. Individualism	.47**	-.45**	-.24	-.80**	.77**	.41**	-.86**	-.10
